# Mechanisms Underlying the Recruitment of Inhibitory Interneurons in Fictive Swimming in Developing *Xenopus laevis* Tadpoles

**DOI:** 10.1523/JNEUROSCI.0520-22.2022

**Published:** 2023-02-22

**Authors:** Andrea Ferrario, Valentina Saccomanno, Hong-Yan Zhang, Roman Borisyuk, Wen-Chang Li

**Affiliations:** ^1^Department of Mathematics and Statistics, University of Exeter, Exeter EX4 4QF, United Kingdom; ^2^School of Psychology & Neuroscience, University of St Andrews, St Andrews KY16 9JP, United Kingdom; ^3^Biorobotics Laboratory, École Polytechnique Fédérale de Lausanne, Lausanne, Switzerland

**Keywords:** integration, interneuron, modeling, recruitment, spinal, swimming

## Abstract

Developing spinal circuits generate patterned motor outputs while many neurons with high membrane resistances are still maturing. In the spinal cord of hatchling frog tadpoles of unknown sex, we found that the firing reliability in swimming of inhibitory interneurons with commissural and ipsilateral ascending axons was negatively correlated with their cellular membrane resistance. Further analyses showed that neurons with higher resistances had outward rectifying properties, low firing thresholds, and little delay in firing evoked by current injections. Input synaptic currents these neurons received during swimming, either compound, unitary current amplitudes, or unitary synaptic current numbers, were scaled with their membrane resistances, but their own synaptic outputs were correlated with membrane resistances of their postsynaptic partners. Analyses of neuronal dendritic and axonal lengths and their activities in swimming and cellular input resistances did not reveal a clear correlation pattern. Incorporating these electrical and synaptic properties into a computer swimming model produced robust swimming rhythms, whereas randomizing input synaptic strengths led to the breakdown of swimming rhythms, coupled with less synchronized spiking in the inhibitory interneurons. We conclude that the recruitment of these developing interneurons in swimming can be predicted by cellular input resistances, but the order is opposite to the motor-strength-based recruitment scheme depicted by Henneman’s size principle. This form of recruitment/integration order in development before the emergence of refined motor control is progressive potentially with neuronal acquisition of mature electrical and synaptic properties, among which the scaling of input synaptic strengths with cellular input resistance plays a critical role.

**SIGNIFICANCE STATEMENT** The mechanisms on how interneurons are recruited to participate in circuit function in developing neuronal systems are rarely investigated. In 2*-*d-old frog tadpole spinal cord, we found the recruitment of inhibitory interneurons in swimming is inversely correlated with cellular input resistances, opposite to the motor-strength-based recruitment order depicted by Henneman’s size principle. Further analyses showed the amplitude of synaptic inputs that neurons received during swimming was inversely correlated with cellular input resistances. Randomizing/reversing the relation between input synaptic strengths and membrane resistances in modeling broke down swimming rhythms. Therefore, the recruitment or integration of these interneurons is conditional on the acquisition of several electrical and synaptic properties including the scaling of input synaptic strengths with cellular input resistances.

## Introduction

Most animals need to execute basic motor functions early in development, and the strength and complexity of movement then increase with age (de Vries et al., 1982; O’Donovan, 1999; Drapeau et al., 2002; Gallahue et al., 2012; Wan et al., 2019). In this process, the developing neuronal circuit needs to maintain existing functions while new populations of neurons are added. An orderly connection of developing neurons with their mature partners and their progressive recruitment in circuit activities is critical. Two basic conditions need to be met; first, the outputs from developing neurons onto the existing circuit should not disrupt circuit functions, and second, the inputs from the existing circuit onto the developing neurons should not be excitotoxic and interrupt their further differentiation. Because the circuit needs to sustain network outputs, we expect appropriate properties must be expressed in the developing neurons to allow this smooth integration.

These properties may belong to intrinsic and firing properties of developing neurons. For example, membrane input resistance (R_inp_; and time constant) decreases when neurons mature in development, e.g., in sensory motor cortex (McCormick and Prince, 1987), prefrontal cortex (Zhang, 2004), amygdala (Ehrlich et al., 2012), and thalamus (Ramoa and McCormick, 1994), and action potentials become narrower (Spitzer and Baccaglini, 1976; Zhang, 2004; Ehrlich et al., 2012), or neurons acquire persistent and hyperpolarization-activated inward currents (Sharples and Miles, 2021). Developing neurons have different synaptic properties from their mature counterparts as well, including the composition of postsynaptic glutamatergic receptors that conveys developmental synaptic plasticity (Isaac, 2003; Herring and Nicoll, 2016) and vesicle release machinery (Mozhayeva et al., 2002; Andreae et al., 2012). How do developing neurons integrate into a functioning circuit? Henneman’s size principle states that as movements become stronger, small motoneurons with high input resistances are recruited before larger ones with lower input resistances (Henneman, 1957; Henneman et al., 1965b; McLean et al., 2007; Gabriel et al., 2011). Further determinants of this type of motor-strength-based recruitment include soma size, intrinsic properties, and synaptic inputs (McLean et al., 2007; Gabriel et al., 2011). This recruitment and decruitment of neurons take place when different motor strength is required or with the expansion of the behavior repertoire (McLean et al., 2007; Fetcho and McLean, 2010; Tripodi and Arber, 2012; Pujala and Koyama, 2019).

Will developmental recruitment follow rules similar to the motor-strength-based size principle? The properties of developing neurons have been compared with those of their adult counterparts mostly *in vitro* (Zhang, 2004; Ehrlich et al., 2012). Few studies have simultaneously monitored network functions and identified the relation between neuronal physiological, anatomic properties and the developmental recruitment. In 2-d-old *Xenopus laevis* tadpoles, we studied the developmental recruitment by analyzing neuronal intrinsic and firing properties, synaptic and anatomic features of inhibitory interneurons in the intact spinal circuit, while simultaneously monitoring network outputs resembling natural swimming behavior (Roberts et al., 2010). We correlated these measurements with cellular input resistances, which have been widely reported to closely reflect how advanced neurons are in development (McCormick and Prince, 1987; Ramoa and McCormick, 1994; Zhang, 2004; Ehrlich et al., 2012). We then used computer modeling to reveal that input synaptic currents during swimming played a key role in the orderly integration of developing neurons in the tadpole swimming circuit.

## Materials and Methods

### Electrophysiology and anatomy

Pairs of adult male and female *Xenopus laevis* were injected with human chorionic gonadotropin to induce mating following procedures approved by the local Animal Welfare Ethics Committee and United Kingdom Home Office regulations. Embryos were then collected and incubated at varying temperatures to stagger their development speeds. Tadpoles at stage 37/38, the sex of which couldn’t be identified, were cut open to allow immobilization for 20–30 min using α-bungarotoxin (12.5 µm, Tocris Bioscience) after brief anesthetization with 0.1% Ethyl 3-aminobenzoate methanesulfonate (MS-222, Catalog #886-86-2, Sigma-Aldrich). The saline included the following (in mm): 115 NaCl, 3 KCl, 2 CaCl_2_, 2.4 NaHCO_3_, 1 MgCl_2_, and 10 HEPES, with pH adjusted to 7.4 with NaOH. After immobilization, the tadpole was fixed onto a rubber stage with pins. Dissections were conducted to expose the nervous system, and ependymal cells lining the central canal of caudal hindbrain and rostral spinal cord were removed to expose neuronal cell bodies to allow whole-cell recordings. Whole-cell recordings were made in either current-clamp or voltage-clamp mode using either an Axon-2B or MultiClamp 700B amplifier. Patch pipettes were filled with 0.1% neurobiotin (Vector Laboratories) in the intracellular solution containing the following (in mm): 100 K-gluconate, 2 MgCl_2_, 10 EGTA, 10 HEPES, 3 Na_2_ATP, and 0.5 NaGTP, adjusted to pH 7.3 with KOH. Fictive swimming was induced by stimulating the tadpole trunk skin using a single 0.5 ms current pulse. Fictive struggling was induced by stimulating the rostral trunk skin repetitively at a frequency between 30 and 40 Hz. Motor nerve (m.n.) recordings were made by placing suction electrodes on the swimming muscle clefts. Loose patch recordings were made using whole-cell recording electrodes containing intracellular pipette solution after applying gentle suction to the somata membrane.

Final identification of neurons was based on their physiology during swimming and struggling and anatomy revealed by neurobiotin staining after recordings, the protocol of which was described previously (Li et al., 2001). The neurobiotin filling of these neurons was examined using a 100× oil immersion lens. Dendrites and somata were hand drawn with the aid of a drawing tube using the 100× oil immersion lens, and the axons were traced with 10× lens. The longitudinal positions of neuronal somata were measured relative to the mid/hindbrain border, and axon trajectories were measured relative to somata. Where multiple ascending or descending branches existed, only the longest one was measured and represented. Most commissural interneurons (cINs) and ascending interneurons (aINs) are unipolar; that is, the axons arise from the primary dendrites (Li et al., 2001). The starting point of an axon was determined by the narrowing of dendrites to an even diameter.

cIN/aIN responses to current injections at rest and during fictive swimming and struggling were recorded in current-clamp mode. Spike threshold was defined as the membrane potential of an action potential when its derivative of derivative peaked; that is, the depolarization accelerated at its highest speed (see Fig. 4*A*). Spike duration was measured as the time difference between the two points when the membrane potential crossed 0 mV. AHP size was the difference between the spike threshold and the AHP trough. Spike height is the difference from the AHP trough to the spike peak. The compound EPSCs and IPSCs they received during swimming were recorded in voltage-clamp mode by clamping the membrane potential at around −60 mV and 0 mV, respectively. Only voltage-clamp recordings with a stable series resistance <30 MΩ (compensation, 70–85%) were used for quantifying synaptic currents.

### Modeling synaptic conductance in paired recordings

In paired recording, leak currents were not subtracted when the postsynaptic cell was recorded in voltage-clamp mode. Synaptic conductance was calculated as the difference between the resting membrane conductance before cIN/aIN spiking and that at the peak/trough of IPSCs. When the postsynaptic cell was recorded in current-clamp mode, synaptic conductance is estimated using multiple compartmental modeling. IPSP reversal was estimated from the regression line on the *I*–*V* plot. The anatomy of the postsynaptic neuron was drawn using a 100× oil lens. The potential synaptic contact locations were also examined at the same magnification. Then the long and short axis of somata, dendrite length, and diameter and distance from synaptic contacts to soma were measured and used as model parameters. Any process with a diameter of <2 µm was omitted to simplify modeling. Postsynaptic neurons typically had one to three main dendrites with one to three synaptic contacts with *en passant* presynaptic axon. In the case of more than one contact, the dendrites were merged as one assuming the same dendrite diameter. Soma was modeled as a single cylindrical compartment and dendrite as 10 compartments in series connection. Specific conductance for the soma and dendrites was given the same value and manually adjusted to match the cellular input resistance (R_inp_) obtained in experiments. Once the R_inp_ was matched, resting membrane potential (RMP), reversal, and measured average IPSP size to a certain current injection level were fed to the model for synaptic conductance optimization. The optimization process matched the IPSP in the model with the IPSP in experiments and returned the corresponding synaptic conductance value by minimizing the squared difference between model and experimentally measured IPSP voltage peaks using the Newton–Raphson method.

### Modeling swimming neuronal network

**Figure 1.**
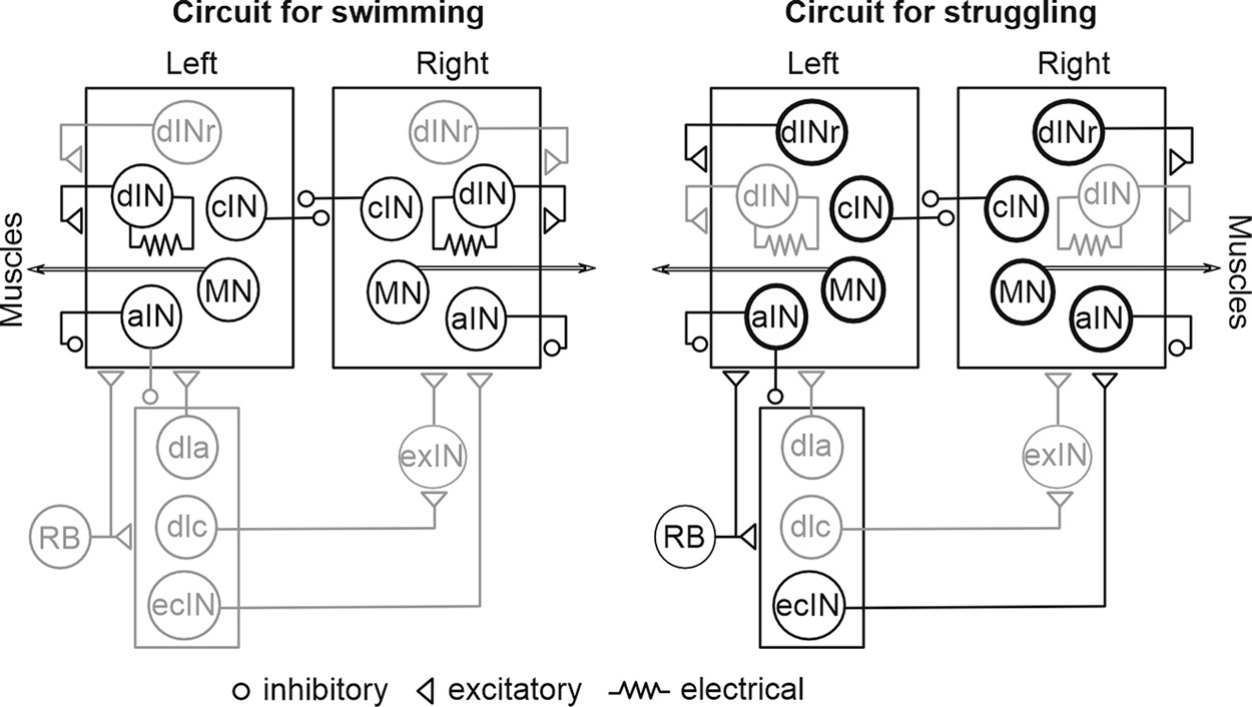
Tadpole cINs, aINs, and their synaptic connections with other spinal/hindbrain neurons (modified from Li, 2015). Black circles stand for activity. Thickened circles denote vigorous activity in struggling. Gray means no/depressed activity during swimming or struggling. Sensory pathway neurons are RB; dla; dlc; ecIN (excitatory commissural interneuron); exIN, hindbrain extension neuron. Other types of neuron active in swimming and struggling rhythms are dINr (repetitive firing dIN); dIN; MN. Each circle represents a population of neurons. Synapse on boxes means all neurons inside receive the input.

**Figure 2.**
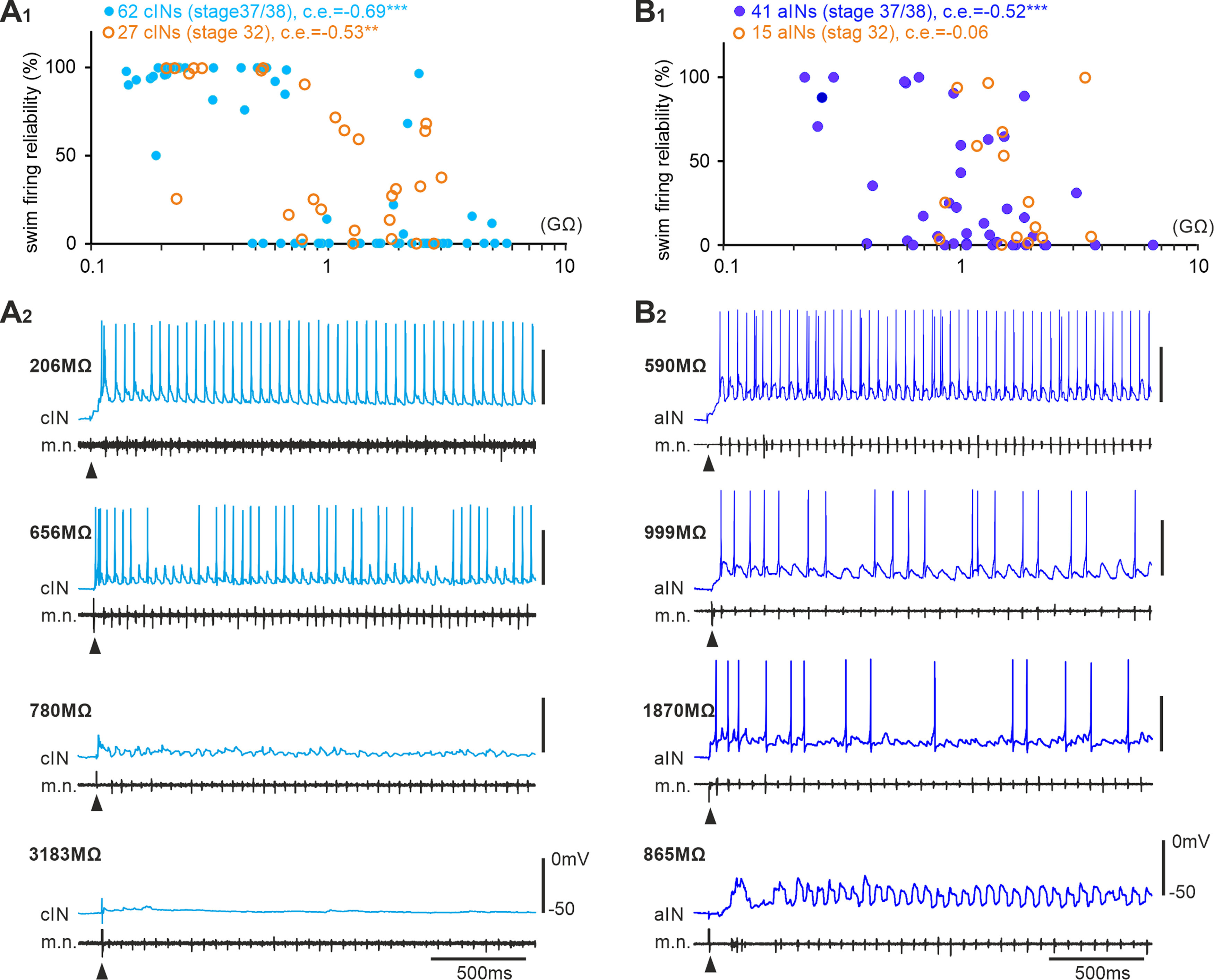
Recruitment order of cINs and aINs in swimming by their R_inp−_. ***A_1_***, ***B_1_***, cIN and aIN firing reliabilities plotted against their R_inp−_. Correlation coefficients (c.e.) with significance levels (***p < 0.001, **p < 0.01) are given above the plots in this and following figures. Filled blue circles are recordings from stage 37/38 tadpoles, and empty orange ones are for recordings from stage 32 embryos. ***A_2_***, ***B_2_***. Examples of cIN and aIN activity in stage 37/38 tadpoles during swimming started by electrical skin stimulation (arrowheads). R_inp−_ of each neuron is indicated on the left side of each recording trace.

The network model contained 1382 single-compartment Hodgkin–Huxley neurons connected by ∼90,000 synapses, modified from previous, biologically realistic models (Sautois et al., 2007; Johnston et al., 2010; Borisyuk et al., 2014; Roberts et al., 2014; Ferrario et al., 2018a; 2021) and resembled a 1.5-mm-long section of the tadpole spinal cord. We simulated the axon growth during development and prescribed synaptic connections at the intersections of axons with dendrites (Borisyuk et al., 2014; Roberts et al., 2014). The general connectivity between neural populations was in line with the schematics in Figure 1. The sensory Rohon–Beard (RB) neurons, dorsolateral commissural interneurons (dlc), and dorsolateral ascending interneurons (dla) had the same composition of membrane ion channels as the motoneurons (MNs) in Dale (1995). aIN and cIN ion channel composition followed Sautois et al. (2007), so they could show delayed firing to current injections (see Fig. 5). As aINs tend to have more dendrites, and thus a larger surface area, than cINs (Li et al., 2001), we modified the capacitance of aIN model from 4 pF to 9 pF. The descending interneuron (dIN) model was based on that used in Hull et al. (2015) but was simplified to a single soma/dendrite compartment, and it exhibited typical dIN rebound firing and oscillatory activity to NMDA perfusion (Roberts et al., 2014; Ferrario et al., 2021). The aIN/cIN R_inp_ was randomly assigned using a generalization procedure (Borisyuk et al., 2014) to match the range and distribution of experimental data in Figure 2, *A1* and *B1*, without the outward rectification properties. In this dataset we excluded values of R_inp_ < 300 MΩ (four aINs/nine cINs), to avoid the use of excessively high excitatory synaptic inputs (which diverges exponentially to ∞ as R_inp_ →0) to drive spiking during swimming. Excitatory synapses in the model had glutamatergic AMPARs and NMDARs with Mg^2+^ voltage-dependency and inhibitory synapses were glycinergic. There was electrical coupling among dINs and MNs (Perrins and Roberts, 1995; Li et al., 2009). Different from previous tadpole models, excitatory synapses from dINs to both aINs and cINs included both AMPAR and NMDAR components measured experimentally (see Fig. 6). aIN synaptic strength and decay time were set to 0.135 nS and 20 ms, respectively to avoid mid-cycle rebound firing in dINs and network synchrony rhythms (Li et al., 2014). cIN inhibition strength was increased from 0.4 nS to 0.7 nS to compensate for the reduction in reliable-firing cINs from our previous models (Roberts et al., 2014).

**Figure 3.**
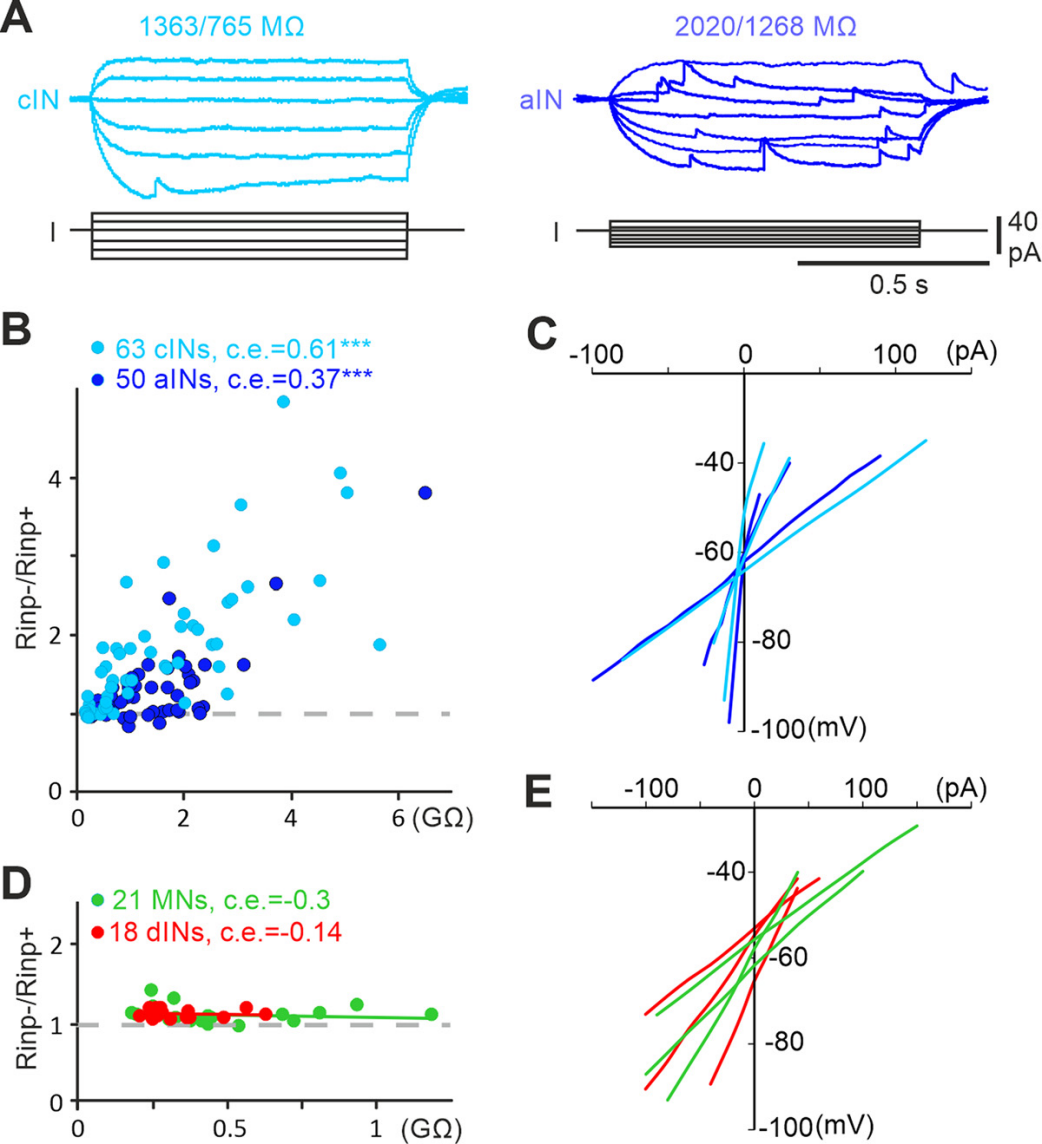
Cellular input resistances of neurons active in tadpole swimming. ***A***, *I*–*V* tests of a cIN and an aIN with rectification using step currents (R_inp−_/R_inp+_ on top of traces). ***B***, R_inp−_/R_inp+_ plotted against R_inp−_. Correlation coefficient (c.e.) and significance (*p < 0.05, ***p < 0.001) are indicated above plots. ***C***, Example *I*–*V* curves for cINs (light blue) and aINs (dark blue) with different R_inp_. ***D***, R_inp−_/R_inp+_ plotted against R_inp−_ for MNs and dINs with little rectification. ***E***, Their *I*–*V* curve examples (MNs, green; dINs, red). Gray dashed lines in ***B*** and ***D*** indicate R_inp−_/R_inp+_ of 1.

We incorporated the negative correlation between cIN/aIN R_inp_ and strengths of input synaptic currents during swimming (see Figs. 6*B1*,*B2*, 7*B*) in the control model and then matched the cIN/aIN firing reliability in modeling with the experimental data in Figure 2, *A1* and *B1*, by applying the following steps: (1) We estimated the synaptic conductance using Ohm’s law, $g_{syn}=I_{syn}/(V_{hold}-V_{rev}),$ where $I_{syn}$ is the measured compound EPSC (tonic, on cycle) or IPSC (early cycle, midcycle; see Figs. 6*B1*,*B2*, 7*B*), $V_{hold}$ is the holding membrane potential and $V_{rev}$ the reversal potential of each synapse (∼0 mV for EPSCs and about −60 mV for IPSCs); (2) we fitted the relation between estimated compound conductance data and R_inp_ using exponentially decaying functions, $f\left(R_{inp}\right)=ae^{-bR_{inp}}$. Python library SciPy (Virtanen et al., 2020) was used to optimize parameters *a* and *b* for each class of conductance (see Fig. 12, blue curves); (3) we estimated unitary synaptic conductance by dividing each compound conductance with the number of active presynaptic neurons in the model (Borisyuk et al., 2014; Roberts et al., 2014), assuming them firing reliably and synchronously on each swimming cycle; and (4) we multiplied the unitary EPSC strengths of cINs/aINs with high R_inp−_ by three to account for the rectification properties (Fig. 3*A*,*B*). As a result, 39% cINs fired spikes (compare 39% in experiments) on >75% cycles, and 27% aINs fired spikes (compare 24% in experiments) on >60% cycles in the swimming rhythms generated by this control model.

We hypothesized that the experimentally derived negative association between cIN/aIN R_inp_ and the input synaptic current strengths (see Figs. 6*B1*,*B2*, 7*B*) was critical for the swimming rhythm generation. To test this hypothesis, we constructed randomized, reversed, and mature inputs models by implementing artificial relations between aIN/cIN R_inp_ and the input synaptic current strengths. In randomized models, we assigned each cIN/aIN a random value of compound synaptic conductance from the data distributions (see Figs. 6, 7) using a generalization procedure (Borisyuk et al., 2014). In the reversed model, we replaced the exponentially decaying dependence in the control model (see Fig. 12, blue curves) by the respective reversed exponentials, $f_{rev}\left(R_{inp}\right)=ae^{b(R_{inp}-R_{max}-R_{min})}$, where $R_{max}$ ( $R_{min}$) is the maximum (minimum) of the $R_{inp}$ values (see Fig. 12, red curves). In addition, the unitary synaptic strengths of postsynaptic neurons with $R_{inp}$ greater than 2.1 *G*Ω were set to the peak value of the reversed exponentials to avoid excessively high values of synaptic strengths. In the mature inputs model, all aIN and cINs were given the strong synaptic inputs as the ones with low R_inp_ received in experiments. In all three models, the distribution of cIN/aIN R_inp_ was kept the same as in the control model. Unitary synaptic conductance was also calculated and scaled up in similar way to what was used in the control model.

In the randomized models, we classified simulation outputs in three groups. Typical swimming rhythms were characterized by periodic activity alternating between the two sides at a frequency between 15 and 19 Hz, similar to the outputs from the control model but in some cases with increased midcycle dIN/cIN spiking. The second group showed one-sided rhythms in which activity persisted only on one side of the network. The frequency of the one-sided activity roughly doubled that for the normal swimming frequency of 15 to 19 Hz, a rhythm likely sustained by dIN postinhibitory rebound firing following the ipsilateral aIN inhibition. The third type of output only showed brief activity in the network, which failed in a couple of rhythmic cycles. The termination of activity was always preceded with tonic firing of many cINs/aINs with high R_inp_ in the network.

To understand how swimming rhythms failed, we analyzed the cIN/aIN spiking phase in the swimming cycle and strengths of their synaptic outputs onto dINs in control, randomized, and reversed models. We used the reliable dIN spiking to determine swimming cycles, and only simulation periods with activities on both sides were used for analyses. The phase of each cIN/aIN spike was calculated as its delay from the preceding dIN spike divided by the cycle period determined by the immediate, corresponding two dIN spikes ( Φ∈[0,1]). The strength of each aIN/cIN spike was the number of its connections to dINs normalized to the maximal connections any aIN/cIN could make to all dINs in the network ( A∈[0,1]), representing the influence of aIN/cIN spiking on dIN firing and swimming rhythm generation. The phases and strengths of all aIN/cIN spikes in individual simulations are shown in circular plots (see Fig. 13*A3*, *B3*, 14*D*). 

### Experimental design and statistical analyses

Data were analyzed using DataView software (from Dr. W. J. Heitler, University of St Andrews) and IBM SPSS Statistics software. Two-tailed Pearson’s correlation was conducted on datasets with normal distributions to examine whether a linear relationship existed between variables. Otherwise, two-tailed Spearman's rank correlation was used to identify monotonic, curvilinear relationships. Independent samples Mann–Whitney *U* tests or Kruskal–Wallis tests were conducted in cases where data were not normally distributed to compare mean ranks or medians. Levene’s test was used to compare variances of cIN/aIN spiking phases in different modeling outputs.

## Results

Different neuronal types and synaptic connections in the *Xenopus* tadpole swimming circuit have been systematically delineated based on physiological, anatomic, neurochemical and pharmacological criteria (Roberts et al., 2010). We focus on two types of inhibitory interneurons active during fictive swimming, cINs and aINs (Fig. 1). Immunostaining for glycine (Dale et al., 1986; Roberts et al., 1988) and GABA (Roberts et al., 1987) has revealed that the cIN and aIN populations keep increasing from when tadpoles start swimming at around stage 32 until they hatch at stage 37/38 (Nieuwkoop and Faber, 1956). As the spinal circuit expands, the basic swimming pattern (i.e., frequency, left-right coordination, burst duration, and rostrocaudal delay) remains unchanged until stage 42 (Sillar et al., 1991). We therefore recorded neurons mainly at stage 37/38, when both mature and developing interneurons coexist, and ask how they were recruited during tadpole swimming.

### Recruitment of cINs and aINs during swimming

When the skin of tadpoles at stage 37/38 is touched briefly, they swim away at a frequency of 10–25 Hz (Roberts et al., 2010). During swimming, tadpole central pattern generator (CPG) neurons fire in a one-spike-per-cycle manner to sustain swimming rhythms after the transient sensory stimulus. To quantify cIN/aIN recruitment during swimming, we measured their firing reliability, that is, percentage of cycles with spikes during the initial 5 s of swimming. In stage 37/38 tadpoles, we found both cIN and aIN firing reliabilities during swimming were negatively correlated with their cellular input resistances (R_inp_), measured with negative step current injections (*p <* 0.001, both Spearman’s rank correlation; Fig. 2). The distribution of cIN firing reliability was skewed either to reliable firing or no firing. In contrast, the distribution of aIN firing is more skewed toward no firing with many neurons firing little or unreliably. The data show an orderly recruitment of cINs and aINs in swimming by their R_inp_.

R_inp_ has been found to decrease with development (McCormick and Prince, 1987; Ramoa and McCormick, 1994; Zhang, 2004; Ehrlich et al., 2012). To confirm whether there was a similar R_inp_ decrease in cINs/aINs in development, we recorded 27 cINs and 15 aINs in younger embryos around stage 32 when stable swimming just started to emerge. There was a negative correlation between cIN R_inp_ and firing reliability (*p <* 0.01, Spearman’s rank correlation), but aIN R_inp_ and firing lacked correlation (Fig. 2). In comparison with neurons in stage 37/38 tadpoles, the cIN R_inp_, cIN, and aIN firing reliabilities were similar, but stage 32 aINs had higher R_inp_ (*p <* 0.05, independent samples Mann–Whitney *U* test). We further divided the neurons into two subgroups, one group with ≥50% firing reliability during swimming and the other with <50% firing reliability. For 13 cINs and 6 aINs with ≥50% firing reliability at stage 32, their R_inp_ was higher than their stage 37/38 counterparts (*p <* 0.05, independent samples Mann–Whitney *U* test; Fig. 2*A1*,*B1*). This confirmed that R_inp_ could be used as an indicator for developmental maturation as in other preparations, at least for neurons recruited to fire reliably during swimming.

### Neuronal intrinsic and spiking properties

Could some intrinsic properties that cINs and aINs possess determine their recruitment during swimming in stage 37/38 tadpoles? We first identified that cINs and aINs showed outward rectification to DC injections around their RMPs, especially when the R_inp_ was high. Neurons with outward rectification requires larger inward currents to get depolarized/excited than outward currents to become inhibited by the same amplitude. We used the ratio of the resistance measured with negative DC (R_inp−_) to that measured with positive DC (R_inp+_) as an index for rectification. Correlation was found between this ratio and R_inp−_ in cINs and aINs (both Spearman’s rank correlation; Fig. 3*A–C*). For comparison, similar correlation is absent in the other two types of neurons active in tadpole swimming, that is, dINs and MNs (both Spearman’s rank correlation; Fig. 3*D*,*E*).

**Figure 4.**
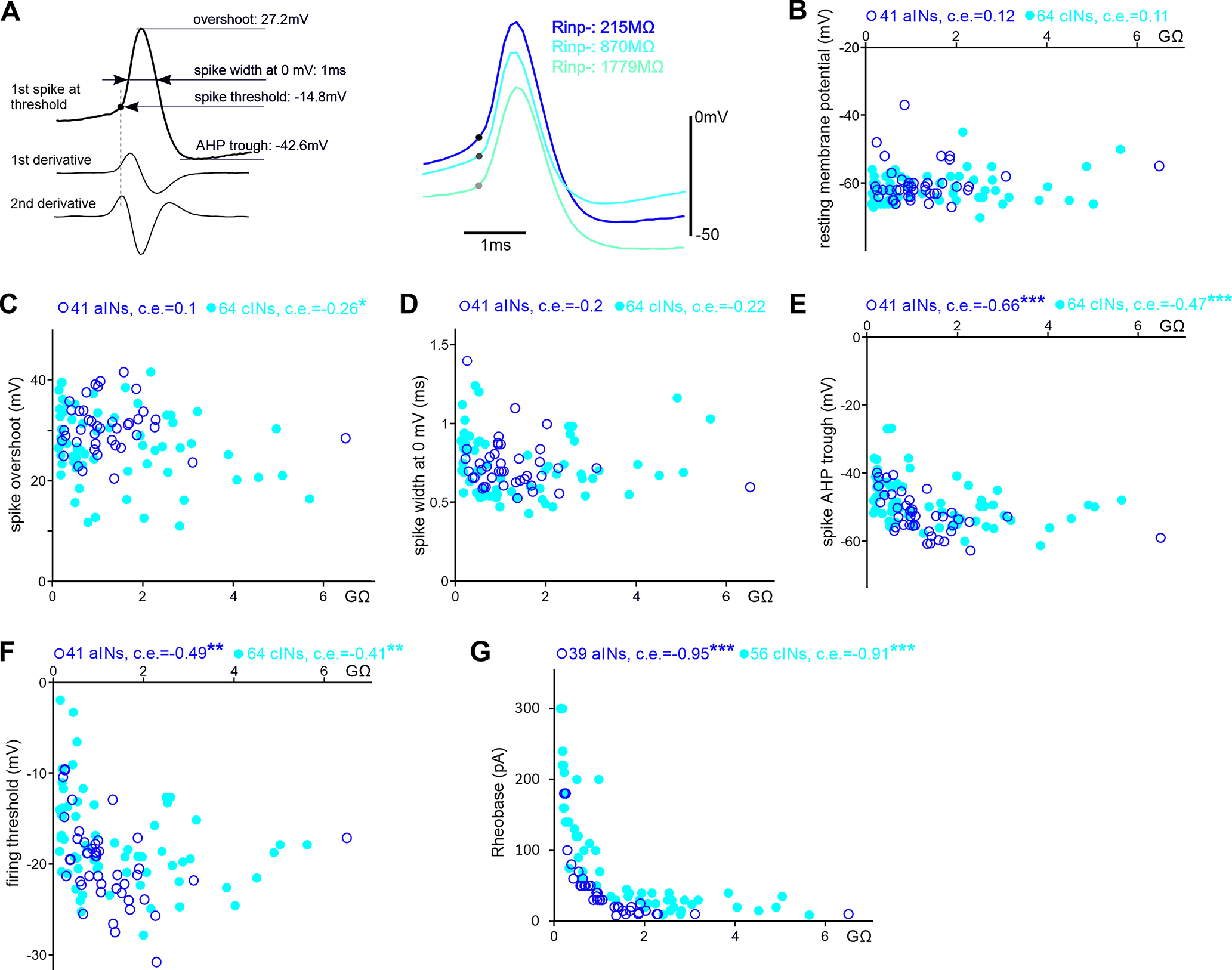
The relation between R_inp−_ and cIN/aIN RMP and spike parameters. **A**, Measuring spike parameters using derivatives, where the second derivative is calculated from the first derivative of the spike trace (left). Dashed line indicates the peak time of the second derivative used to determine the spike threshold (filled circle). Spike width is measured between time points when the membrane potential crosses 0 mV. Spikes from three cINs with different R_inp−_ (right, color coded with text, filled circles represent thresholds). ***B–G***, The relation between R_inp−_ of cINs/aINs and their RMP, spiking overshoots, spike widths, AHP troughs, thresholds and Rheobases (all Spearman’s rank correlation, significance *p < 0.05, **p < 0.01, ***p < 0.001).

We next looked at the relation between R_inp−_ and RMPs and spiking parameters (Fig. 4*A*) of cINs and aINs. In 41 aINs and 64 cINs, spike overshoots were lower in cINs with higher R_inp−_ (*p <* 0.05). Both firing thresholds and spike AHP troughs were negatively correlated with R_inp−_ (AHP, *p <* 0.001; Fig. 4*E*; thresholds, *p <* 0.01; Fig. 4*F*), whereas the RMP and spike width were not (Fig. 4*B*,*D*). There was also a correlation between neuronal rheobases and R_inp−_ (*p <* 0.001; Fig. 4*G*), suggesting cINs/aINs with low R_inp−_ require large synaptic currents to drive their firing during network activity like swimming.

### Firing pattern to current injections

**Figure 5.**
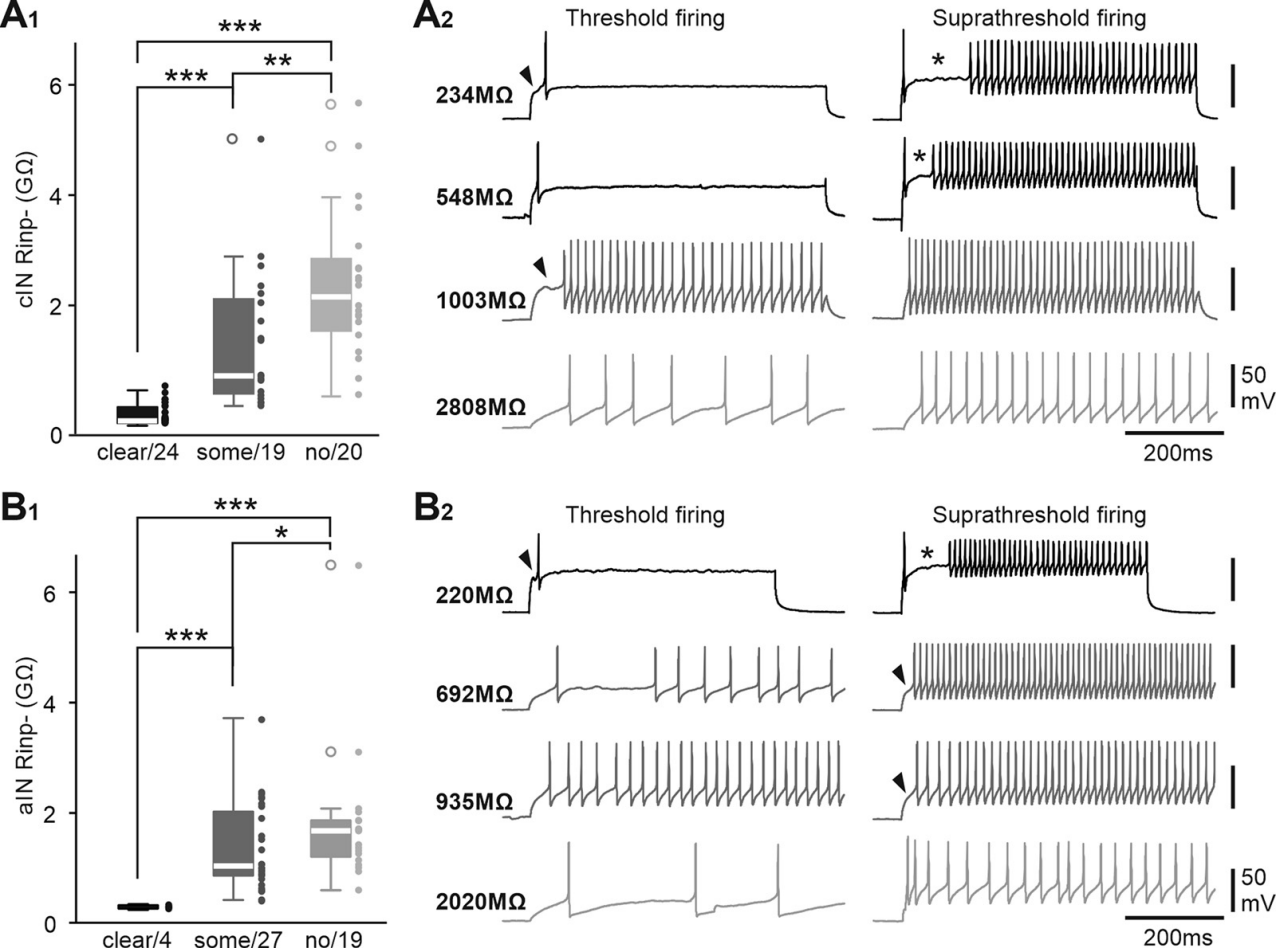
The relation between R_inp−_ and delayed firing in cINs and aINs. ***A_1_***, ***B_1_***, R_inp−_ of cINs and aINs categorized with clear, some, or no delay in their firing to +DC injections; *p < 0.05, **p < 0.01, ***p < 0.001. ***A_2_***, ***B_2_***, Examples of firing patterns from cINs/aINs with different R_inp−_ evoked by threshold and suprathreshold current injections_._ Arrowheads point at delay before the first spike. Asterisk denotes gap between the first and subsequent spikes.

We previously showed that the presence of transient potassium currents (I_A_) in many neurons can cause delay in the onset of spiking, leave a gap in repetitive firing, and affect neuronal firing thresholds (Li, 2015). The negative correlation between R_inp−_ and thresholds suggested that aINs/cINs with lower R_inp−_ could possess I_A_ and show delayed firing to current injections. The aIN and cIN firing were examined with threshold and suprathreshold +DC currents, and the responses were grouped into three categories, delayed firing with a clear gap between the first and subsequent spikes (clear delay), delayed firing from the DC onset (some delay), and no clear delay. The average R_inp−_ of neurons with clear delays was the lowest, and that for neurons with no delay was the highest (*n =* 63 cINs, *p <* 0.001; *n =* 50 aINs, *p* < 0.01; independent samples Kruskal–Wallis tests; Fig. 5).

### Input synaptic currents in cINs and aINs during swimming

The spiking of neurons is determined by their intrinsic properties and also by the synaptic currents they receive. The size principle suggests that neurons with higher R_inp_ should be recruited to fire more reliably in response to similar synaptic currents than those with lower R_inp_. As no pacemaker properties have been identified in aINs or cINs (Li et al., 2010), the inverted recruitment order by R_inp_ suggests they may receive synaptic inputs with strengths scaled with R_inp_. We therefore analyzed the strengths of synaptic currents received by cINs and aINs during swimming.

**Figure 6.**
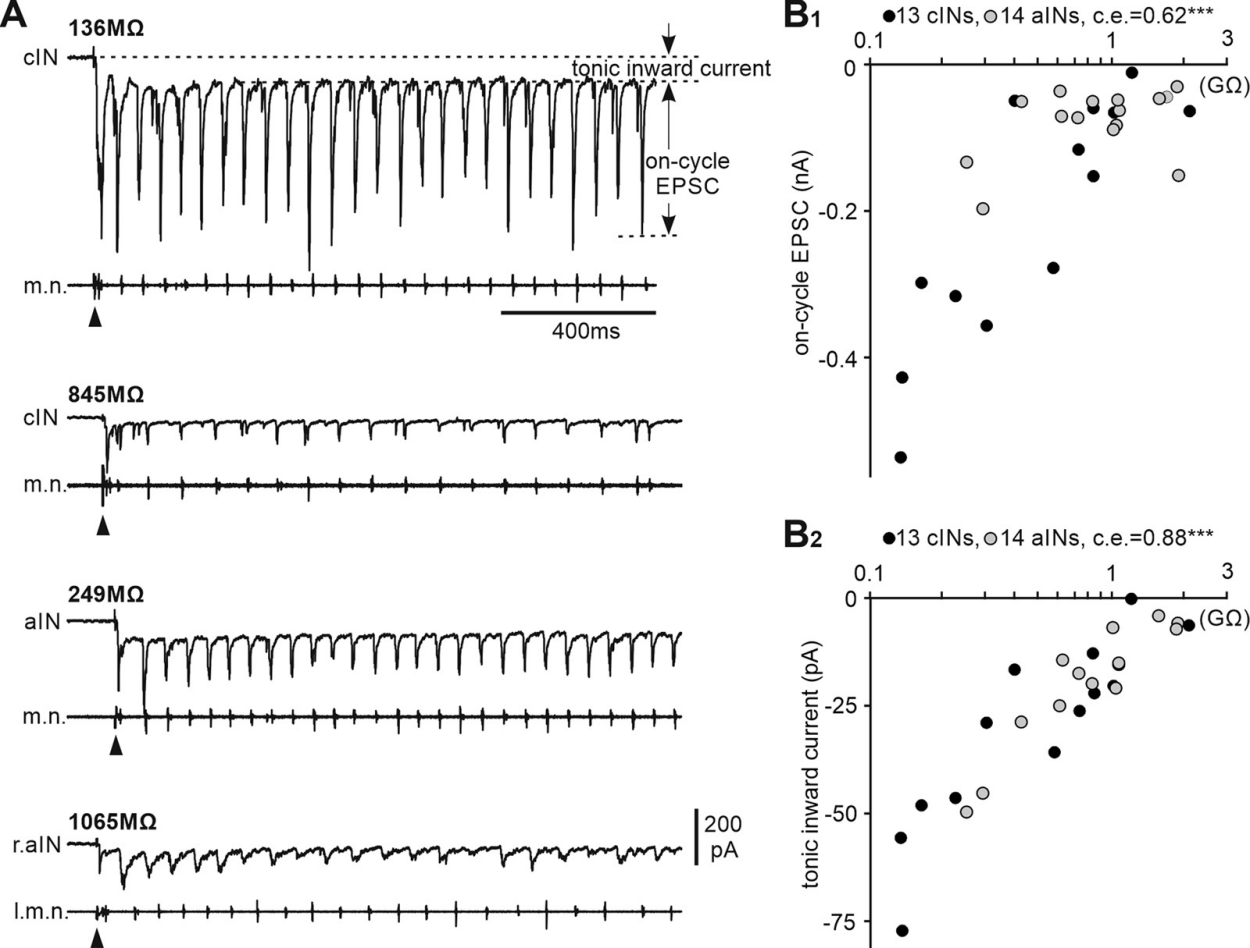
Inward currents that cINs and aINs receive during swimming and their correlation with R_inp−_. ***A***, Examples of tonic inward currents and on-cycle EPSCs in cINs and aINs with indicated R_inp−_. ***B_1_*_,_*_2_***, Correlation between inward currents and R_inp_ (significance ***p < 0.001).

During tadpole swimming, rhythmic firing of CPG neurons including cINs/aINs is driven by the excitatory dINs on the same side. dIN excitation contains a phasic AMPAR- and nAChR-mediated component, which directly drives most CPG firing, and the long-lasting NMDAR-mediated tonic component, which is critical for maintaining swimming rhythms (Li et al., 2004, 2006). To measure dIN-mediated EPSCs we clamped cIN and aIN membrane potentials around −60 mV to reveal the inward tonic NMDAR-mediated currents and phasic on-cycle EPSCs. There was positive correlation between on-cycle EPSC amplitude, tonic inward currents, and the R_inp−_ of a combined dataset of 13 cINs and 14 aINs (Fig. 6*A*,*B*). Regarding the reliability of inward currents, only one cIN and one aIN did not receive measurable tonic inward currents. Similarly, most neurons received 100% reliable on-cycle EPSCs during swimming, except for one cIN (14% with R_inp−_ of 1212 MΩ) and one aIN (18.5% with R_inp−_ of 1571 MΩ). The ratios between on-cycle EPSC and tonic inward current were not correlated with cIN/aIN R_inp−_ (*p =* 0.68, *n =* 12 cINs, 13 aINs; two-tailed Spearman's rank correlation), suggesting a similar excitatory receptor current composition across all cINs/aINs with different R_inp−_.

**Figure 7.**
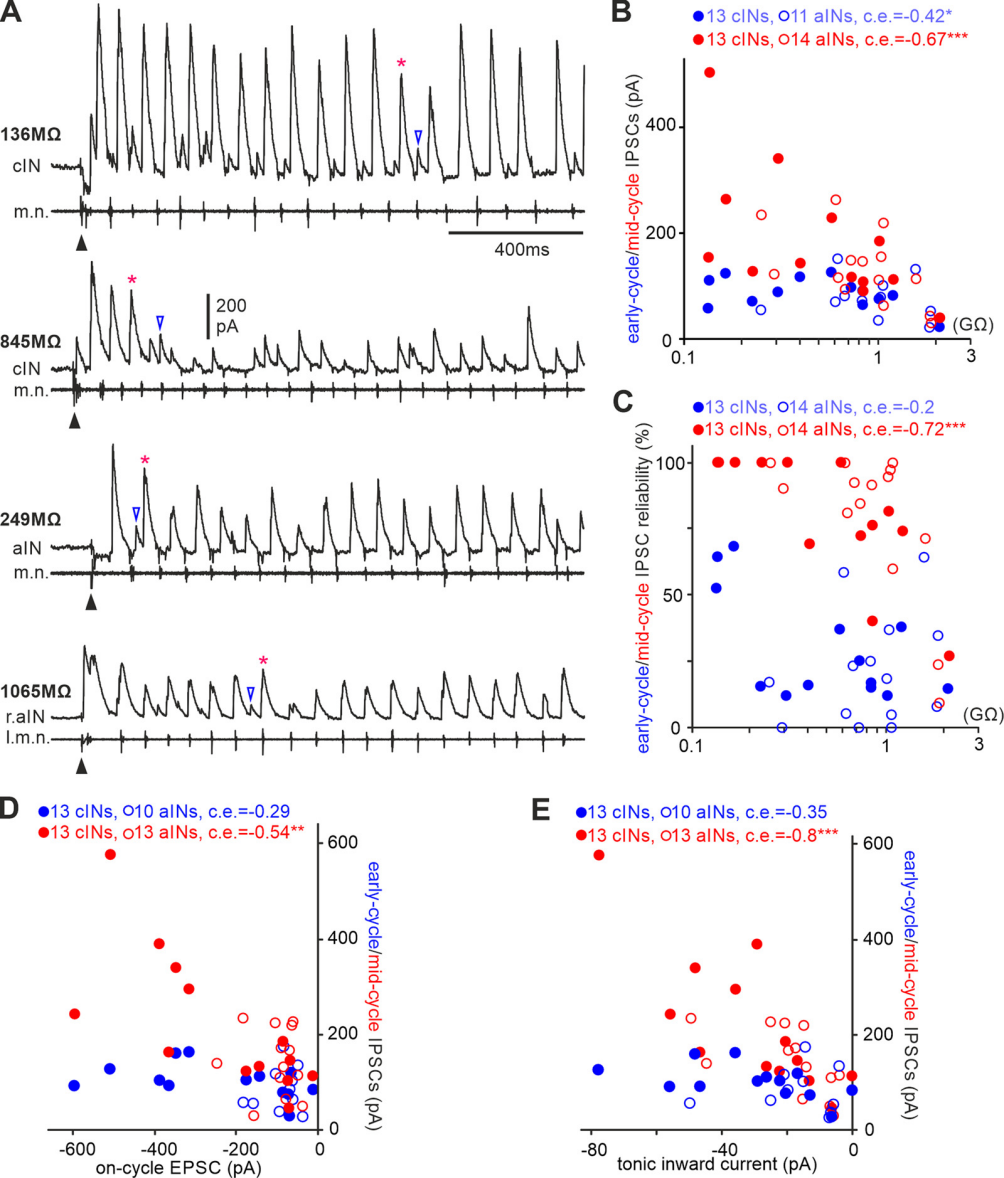
Correlation between IPSCs that cINs and aINs receive during swimming and their R_inp−_ and inward currents. ***A***, Examples of midcycle (*) and early-cycle IPSCs (empty triangles) in cINs and aINs with indicated R_inp−_. Filled triangles indicate time of electrical stimulation starting swimming. ***B***, Correlation between the IPSC amplitude and R_inp−._ ***C***, There lacks correlation between early-cycle IPSC reliability and R_inp−_, but midcycle IPSC reliability is correlated with R_inp−_. ***D***, Midcycle IPSCs are correlated with on-cycle EPSCs, but early-cycle IPSCs are not. ***E***, Midcycle IPSCs are correlated with tonic inward currents but early-cycle IPSCs are not. Correlation significance in ***B–E***, *p < 0.05, **p < 0.01, ***p < 0.001. All are Spearman’s rank correlation except for the relation between early-cycle IPSC and aIN R_inp−_ in ***B*** (Pearson’s correlation).

In addition to excitation, spinal inhibitory neurons also receive midcycle inhibition from cINs on the opposite side and early-cycle inhibition from aINs on the same side (Roberts et al., 2010; Li and Moult, 2012). To measure the two types of IPSCs in the combined dataset of 13 cINs and 14 aINs, we held membrane potentials at ∼0 mV to minimize EPSCs. There was negative correlation between midcycle and early-cycle IPSC amplitudes and the R_inp−_ of these cINs and aINs (Fig. 7*A*,*B*). Midcycle IPSC reliability during swimming (percentage of cycles with midcycle IPSCs) was also negatively correlated with cIN and aIN R_inp−_, suggesting potential differences in their synaptic release probabilities. However, there was no correlation between early-cycle IPSC reliability and cIN and aIN R_inp−_ (Fig. 7*C*).

We also asked whether the different types of synaptic currents were correlated with each other, that is, if they were scaled together or independently regulated. The amplitudes of midcycle IPSCs were correlated with on-cycle EPSCs and tonic inward currents, but such correlation was not observed for the early-cycle IPSCs (Fig. 7*D*,*E*). These data show that the majority of synaptic currents received by cINs and aINs during swimming are scaled with their R_inp−_.

### Synaptic outputs of cINs and aINs

**Figure 8.**
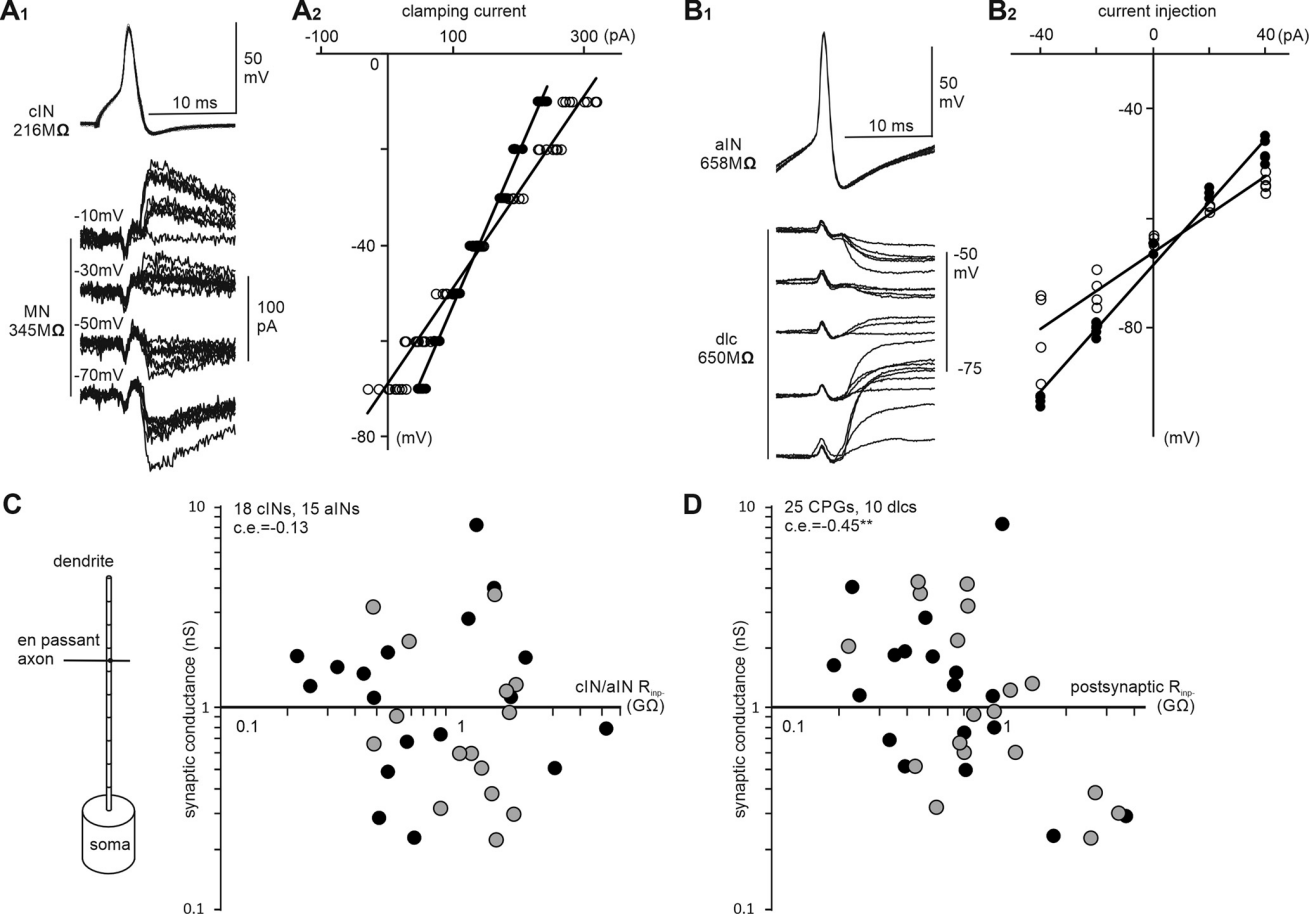
The conductance of cIN and aIN IPSPs/IPSCs in paired recordings is correlated with the R_inp−_ of the postsynaptic neurons. ***A_1_***, Superimposed traces of cIN synaptic currents recorded in an MN in a paired recording, where the presynaptic cIN is recorded in current-clamp mode, and the postsynaptic MN is voltage clamped at four different levels. ***A_2_***, *I*–*V* measurements of the MN in ***A_1_*** at the time before cIN spiking (solid circles) and at the peak/trough of cIN IPSCs (unfilled circles). ***B_1_***, Example traces of aIN unitary IPSPs in a dlc in a paired recording when both the presynaptic aIN and postsynaptic dlc are recorded in current-clamp mode. ***B_2_***, *I*–*V* measurements at the time before aIN spiking (solid circles) and at the peak/trough of aIN IPSPs (unfilled circles) in ***B_1_***. Regression lines in ***A_2_*** are used to estimate conductance at rest and the peak/trough of IPSCs with their difference representing the synaptic conductance. IPSC/IPSP reversal is the point where regression lines in ***A_2_*** and ***B_2_*** (unfilled circles) intersect the vertical axis. ***C***, There lacks correlation between cIN and aIN R_inp−_ and their output synaptic conductance (Spearman’s rank correlation). Diagram shows the simplified multicompartment model used for estimating the conductance of a synapse. ***D***, cIN and aIN output synaptic conductance is correlated with the R_inp−_ of the postsynaptic neuron (Spearman’s rank correlation, **p < 0.01). Solid circles in ***C***, ***D*** are for cINs, and gray ones for aINs as the presynaptic neurons.

Once neurons are recruited to fire action potentials, their contribution to the network will be determined by their output synaptic strength relative to the postsynaptic R_inp_. cINs and aINs are inhibitory, so we could measure the size of IPSPs/IPSCs they produced in the postsynaptic neuron in paired recordings. However, the amplitude of IPSPs/IPSCs is determined by their reversal, which varied considerably in the recordings (range, −38 to −75 mV), although the same pipette solution was used. We decided to analyze synaptic conductance instead. When the postsynaptic cell was recorded in voltage-clamp mode (*n =* 5 cINs), leak currents were not subtracted during the recordings. Synaptic conductance was calculated as the difference between membrane conductance at rest before cIN/aIN spiking and that at the peak/trough of IPSCs (Fig. 8*A*). When the postsynaptic cell was recorded in current-clamp mode (*n* =13 cINs, 18 aINs; Fig. 8*B*), we estimated peak synaptic conductance by using multiple compartment modeling to optimally match IPSPs in paired recordings after reproducing the anatomic feature of the postsynaptic neuron and synapse location (see above, Materials and Methods). There was no correlation between cIN and aIN R_inp−_ and their output synaptic conductance. However, there was correlation between their output synaptic conductance and the postsynaptic R_inp−_ (*p* < 0.01; Fig. 8*C*,*D*). These data show that cIN and aIN output synaptic strengths are scaled to the R_inp−_ of their postsynaptic target cells, not to their own R_inp−_.

### Estimating the number of unitary synaptic currents cINs/aINs received on each swimming cycle

Figure 7 shows that cINs and aINs with higher R_inp−_ received smaller compound synaptic inputs during swimming. The scaling of unitary IPSCs with the postsynaptic neuronal R_inp−_ in Figure 8 provides one possible explanation for reduced compound synaptic inputs in neurons with higher R_inp−_. The number of unitary synaptic currents can also directly determine the amplitude of compound synaptic inputs and influence cIN/aIN recruitment. We used a method similar to the one in Raastad et al. (1996) to estimate the average number of unitary IPSCs/EPSCs that cINs/aINs received on each swimming cycle but without extrapolating the number of undetectable events.

**Figure 9.**
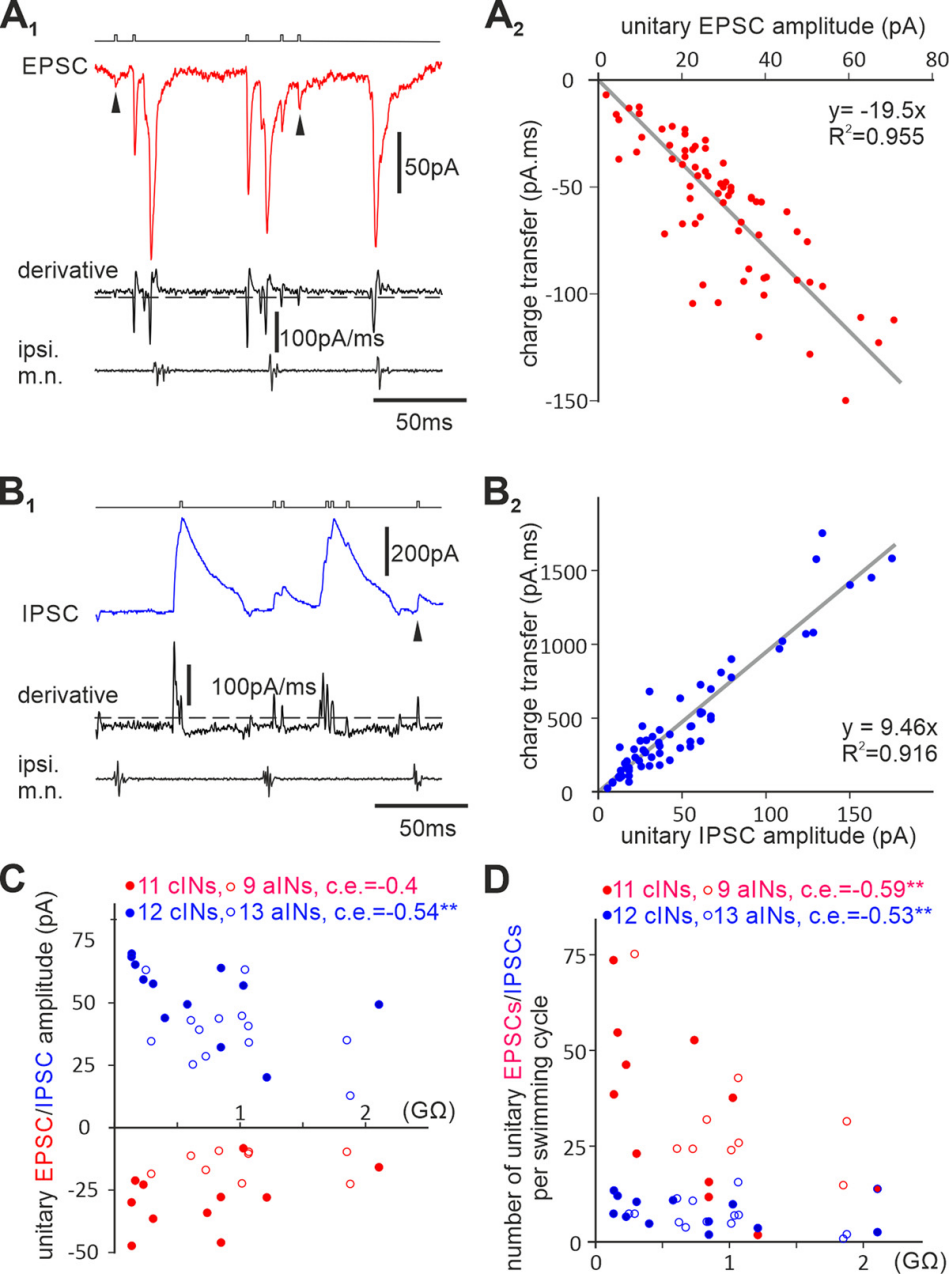
Estimating average numbers of unitary synaptic currents cINs/aINs receive on each swimming cycle. ***A_1_***, EPSCs in a cIN during three swimming cycles and their derivatives used for identifying potential unitary EPSCs (steps in the event channel above). ***A_2_***, Linear regression between unitary EPSC amplitudes and their charge transfers (gray line, *y* = −19.5×, *R*^2^ = 0.955). ***B_1_***, IPSCs in another cIN during two swimming cycles and their derivatives used for identifying unitary IPSCs (steps in the event channel above). ***B_2_***, Linear regression between unitary IPSC amplitudes and their charge transfers (gray line, *y* = 9.46×, *R*^2^ = 0.916). ***C***, Correlation between cIN/aIN R_inp−_ and the unitary EPSC/IPSC amplitudes. ***D***, Correlation between cIN/aIN R_inp−_ and the deduced number of unitary EPSCs/IPSCs they receive on each swimming cycle. In ***A_1_*** and ***B_1_***, dashed lines illustrate thresholds for event triggering, and arrowheads point at lone unitary events used for integrating charge transfer in ***A_2_*** and ***B_2_***. In ***C*** and ***D*,** Spearman’s rank correlation is used for cINs, and Pearson correlation is used for aINs; **p < 0.01.

First, we generated the derivative trace of the synaptic currents with fast onsets of IPSCs/EPSCs producing peaks/troughs. Then a threshold was set in the derivative to pick up potential unitary IPSCs/EPSCs with further manual sorting to exclude highly synchronized, compound on-cycle EPSC or midcycle IPSC events. The amplitude of these potential unitary synaptic currents was measured and averaged (*n =* 76 ± 32.6 events per cell). Lone synaptic events (Fig. 9*A1*,*B1*, arrowheads) were used to measure unitary charge transfers by integrating currents over the EPSC/IPSC duration. Linear regressions were used to estimate the relation between the unitary IPSC/EPSC amplitude and charge transfer (*n =* 64 IPSCs and 68 EPSCs; Fig. 9*A2*,*B2*). Then the total charge transfer by all IPSCs/EPSCs over 10–30 swimming cycles was measured in each cIN/aIN and divided by the number of cycles and unitary charge transfer predicted by the average unitary synaptic currents in that neuron with the regression equations. This allowed us to estimate how many unitary IPSCs/EPSCs every cIN/aIN received during each swimming cycle. Early-cycle and midcycle IPSCs are not discriminated in this analysis for simplicity.

In conformity with Figure 8*D*, the unitary IPSC amplitude was correlated with cIN/aIN R_inp−_ (*n =* 12 cINs, 13 aINs, *p <* 0.01). In contrast, similar correlation between unitary EPSC amplitudes and cIN/aIN R_inp−_ was not significant (*n =* 11 cIN, 9 aINs, *p =* 0.083; Fig. 9*C*). The average number of unitary EPSCs cINs/aINs received on each swimming cycle was negatively correlated with cIN/aIN R_inp−_ (*n =* 11 cINs, 9 aINs, *p <* 0.01). Similar correlation existed for unitary IPSCs (*n =* 12 cINs, 13 aINs, *p <* 0.01, both two-tailed Pearson correlation; Fig. 9*D*). These results suggest that neurons with high R_inp−_ receive smaller numbers of presynaptic input, assuming most unitary synaptic events that contributed significantly to cIN/aIN activity during swimming have been identified.

### Correlation of cIN and aIN anatomy with their firing reliability in swimming

We next asked whether any of cIN and aIN anatomic features could be predictors of their recruitment in swimming. The whole-mount slides of the tadpole CNS allowed us to trace the whole dendritic arbor and axons to their growth cones in the majority of neurons with neurobiotin staining. We measured the longitudinal location of somata, soma area, primary dendrite diameter at its base, primary dendrite length, total dendritic lengths, ascending and descending axon lengths, and combined axon lengths and correlated them with the firing reliability in swimming.

**Figure 10.**
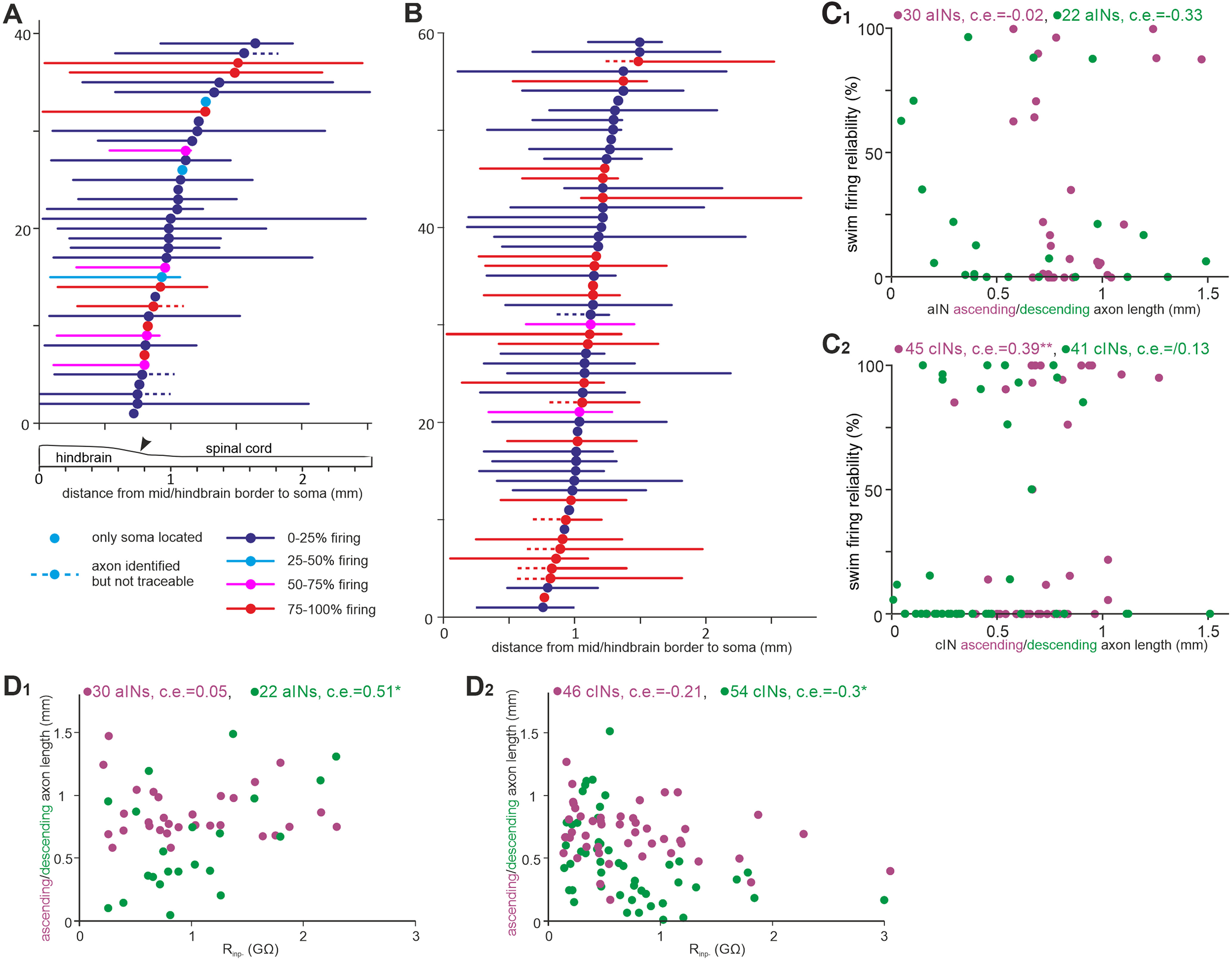
The correlation between cIN and aIN axon lengths and their firing in swimming and R_inp−_. ***A***, The longitudinal location of aIN somata (filled circles) and their simplified, maximal axon trajectories (lines color coded by their firing reliability range) relative to the mid/hindbrain border (0, arrowhead on diagram below indicates obex). ***B***, Location of cIN somata and their axon trajectories (same symbols and color coding as in ***A***). ***C_1_***, aIN firing reliability plotted against ascending and descending axon lengths. ***C_2_***, cIN firing reliability in swimming plotted against their ascending and descending axon lengths. ***D_1_***, aIN R_inp−_ is correlated with their descending but not ascending axon lengths. ***D_2_***, cIN R_inp−_ is correlated with their descending but not ascending axon lengths. Purple text and symbols are for ascending axons, and green ones are for descending axons in ***C_1_*_,_*_2_*** and ***D_1_*_,_*_2_***. All are Spearman’s rank correlation except for the relation between aIN descending axon length and R_inp−_ in ***D_1_*** (Pearson’s correlation, significance *p < 0.05, **p < 0.01).

First, there was no correlation between the longitudinal cIN or aIN soma location and their firing reliability in swimming. For soma and dendritic measurements, only the total aIN dendritic length was negatively correlated with aIN firing reliability (*n =* 29, *p <* 0.01; Table 1). Neither ascending nor descending axon length of aINs was correlated with their firing reliability, although those with shorter ascending axons tended to fire more reliably in swimming (*n =* 22, *p =* 0.14; Fig. 10*A*,*C1*). For cINs, neurons that fired more reliably during swimming had longer ascending axons (*n =* 39, *p <* 0.01), and their descending branches also tended to be longer (*n =* 41, *p =* 0.13; Fig. 10*B*,*C2*, all Spearman’s rank correlations; Table 2). These data suggest axon lengths may be potential predictors of cIN and aIN recruitment in swimming.

**Table 1:** Correlation of aIN anatomic measurements with the main physiology indices

Physiology parameters	Soma location	Soma area	Primary dendrite diameter	Primary dendrite length	Total dendrite length	Ascending axon length	Descending axon length	Combined axon length
R_inp−_	−0.25 (39)	*0.15* (*29*)	0.20 (29)	0.45* (*29*)	0.44* (*29*)	−0.05 (30)	0.47* (*26*)	*0.42* (*22*)
Swimming firing reliability	0.18 (39)	−0.15 (29)	−0.35 (29)	−0.3 (29)	−0.56** (29)	−0.02 (30)	−0.45* (26)	−0.24 (22)
Spikes per struggling cycle	0.16 (25)	*0.06* (*16*)	−0.29 (16)	*0.11* (*16*)	*−0.22* (*16*)	−0.05 (19)	*−0.06* (*16*)	*0.08* (*14*)

Italic indicates Pearson’s correlation; others are Spearman’s rank correlation; sample sizes are in parentheses. Asterisks and boldface signify correlations with statistical significance (*p < 0.05, **p < 0.01).

**Table 2:** Correlation of cIN anatomic measurements with the main physiology indices

Physiology parameters	Soma location	Soma area	Primary dendrite diameter	Primary dendrite length	Total dendrite length	Ascending axon length	Descending axon length	Combined axon length
R_inp−_	0.15 (61)	−0.19 (53)	−0.14 (55)	−0.06 (55)	−0.24 (55)	−0.36* (51)	−0.22 (57)	−0.71** (43)
Swimming firing reliability	−0.23 (59)	−0.01 (52)	−0.13 (54)	−0.03 (54)	−0.04 (54)	0.44** (49)	−0.05 (55)	0.33 (41)
Spikes per struggling cycle	−0.07 (48)	−0.05 (42)	−0.08 (44)	0.26 (44)	0.33 (44)	−0.25 (39)	0.54 (45)	0.5** (32)

All used Spearman’s rank correlation; sample sizes are in parentheses. Asterisks and boldface signify correlations with statistical significance (*p < 0.05, **p < 0.01).

We also examined the relation between cIN and aIN anatomy and their R_inp−_ as neurons with larger dendritic arbors and somata normally have lower R_inp_ in mature circuits. There was no correlation between the longitudinal soma positions of cINs or aINs and their R_inp−_. aIN R_inp−_ was positively correlated with their primary and total dendritic lengths (*n =* 29, *p <* 0.05), opposite to what was expected in mature neurons. For axons, there was positive correlation between aIN descending axon lengths and aIN R_inp−_ (*n =* 22, *p <* 0.05). In contrast, the correlation between cIN descending axon lengths and their R_inp−_ was negative (*n =* 54, *p <* 0.05). The ascending axon lengths were not correlated with R_inp−_ in either type of neuron (Fig. 10*D1*,*2*, Tables 1, 2).

### cIN and aIN activity during struggling

**Figure 11.**
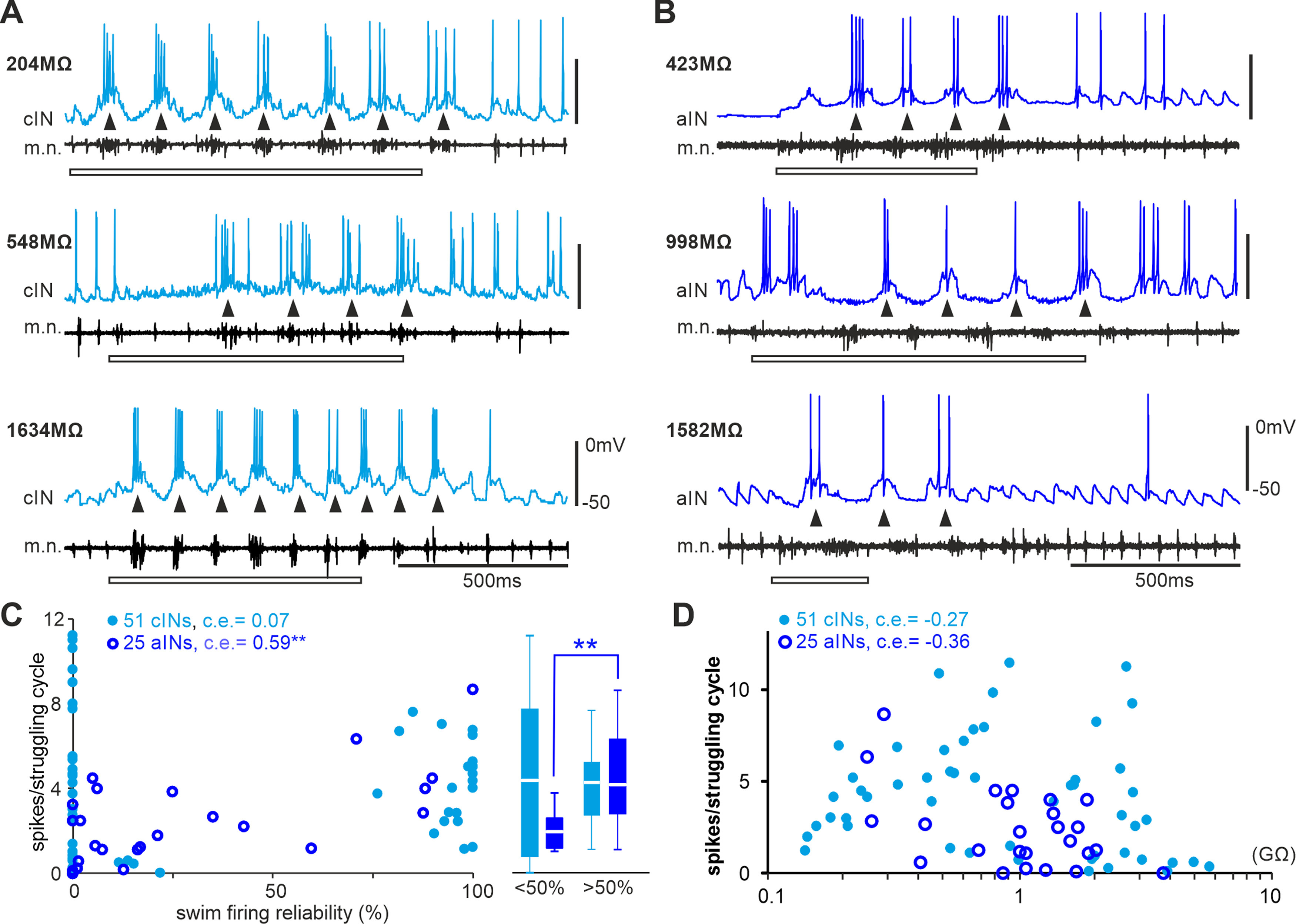
Lack of correlation between cIN and aIN firing intensity during struggling and their R_inp−_. ***A***, ***B***, Examples of cIN (***A***) and aIN (***B***) activity during struggling (started by 30–40 Hz electrical skin stimulation, hollow bars). R_inp−_ of each neuron is given near its recording trace. Arrowheads point at individual struggling cycles. ***C***, Correlating cIN and aIN firing reliability in swimming with their spiking in struggling. Box plots show spikes per struggling cycle of cINs and aINs with < and > 50% firing reliability in swimming. ***D***, Spikes per struggling cycle plotted against cIN/aIN R_inp−_. All are Spearman’s rank correlation (***C***, ***D***; **p < 0.01).

Could cINs or aINs that are inactive in swimming be specialized in struggling activity; that is, is there motor-pattern-based recruitment? When tadpoles are held, they produce stronger and slower contractions at 2–10 Hz called struggling (Roberts et al., 2010). We previously showed that most tadpole swimming CPG neurons were also active during struggling, which could be evoked by stimulating the skin of immobilized tadpoles repetitively (Fig. 11*A*,*B*). The distribution of cIN activity during swimming looked bimodal (Fig. 2*A1*). Neurons typically fire a single spike on each swimming cycle but multiple spikes on each struggling cycle (Berkowitz et al., 2010). We used the number of spikes per struggling cycle as an index for recruitment in struggling as most neurons fire multiply on each cycle.

There was no correlation between firing reliability of cINs in swimming with their spiking in struggling, but for aINs, neurons that fired more reliably in swimming also fired more spikes per struggling cycle (*n =* 25, *p <* 0.01, Spearman’s rank correlation). We separated cINs and aINs into two groups, one group with >50% firing reliability in swimming and the other ≤50%. When the activity of the two groups of neurons in struggling was compared, aINs but not cINs with >50% firing during swimming also fired more spikes per struggling cycle than those aINs with ≤50% firing in swimming (independent samples Mann–Whitney *U* test, *p <* 0.01; Fig. 11*C*). During struggling, there was no correlation between the number of spikes per struggling cycle and R_inp−_ for either cINs or aINs (Fig. 11*D*). The analyses thus did not support a clear segregation of cINs and aINs with specific involvement in struggling.

### Destroying the negative association between the R_inp_ and input synaptic currents broke down swimming in modeled swimming networks

Based on the detailed analyses of cIN/aIN intrinsic and firing properties, their input and output synaptic properties, their anatomy, and their activity during swimming and struggling, it appeared that the input synaptic strengths cINs/aINs received during swimming was a determinant for their recruitment. We next used populational modeling to investigate how altering the experimentally identified negative association between R_inp_ and input synaptic currents affected cIN/aIN activity and the network outputs.

**Figure 12.**
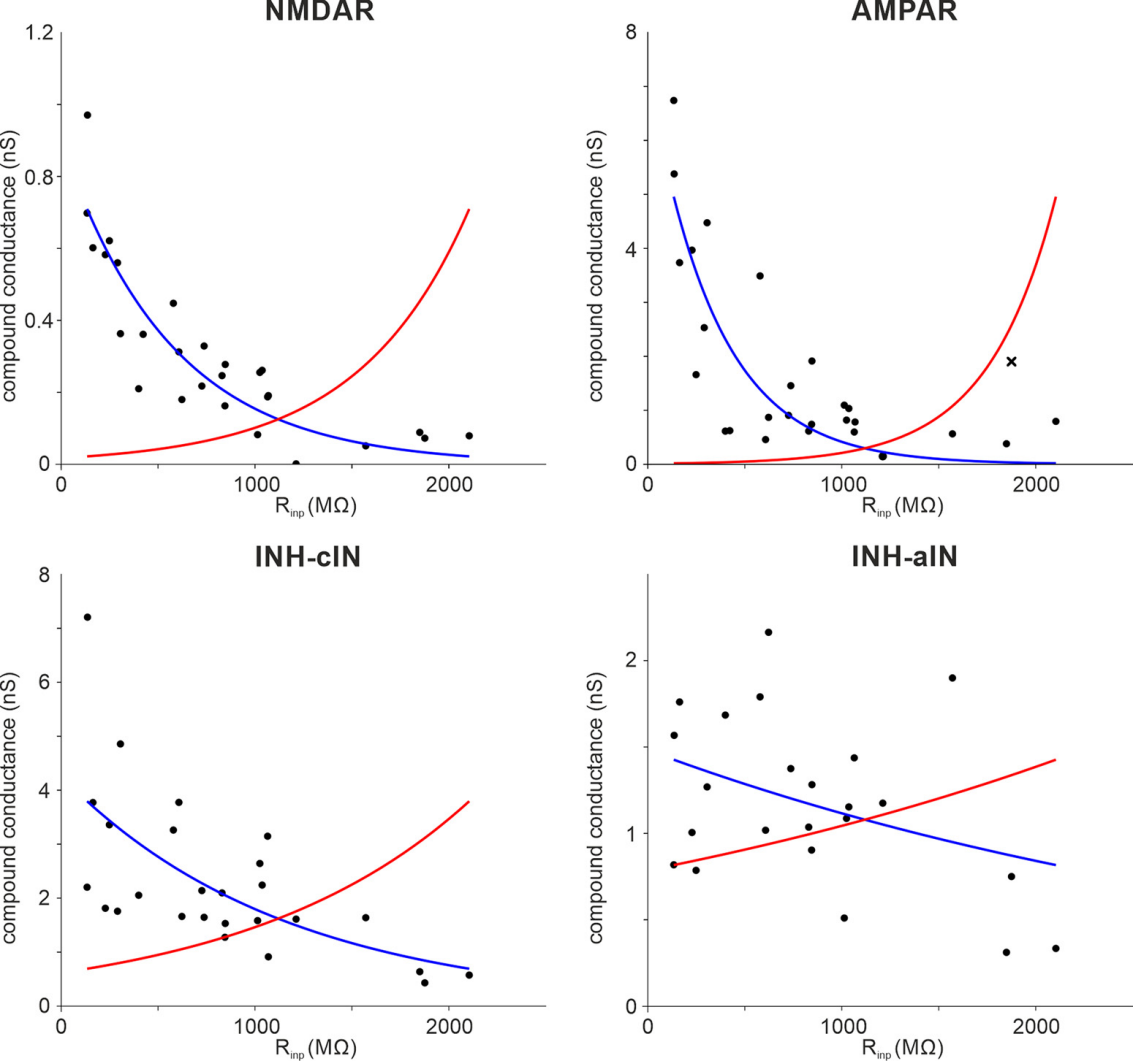
Compound conductance for tonic (NMDAR), on-cycle (AMPAR) EPSCs, midcycle (INH-cIN), and early-cycle (INH-aIN) IPSCs of cINs/aINs during swimming (combined from Figs. 6*B1*_,_*B2*, 7*B*). Blue curves are the best exponential fits for the data used in control models, whereas red curves are reversed exponentials of the blue curves used in the reversed models. Top right, One datum point (*x*) was treated as an outlier and was excluded to achieve better fitting. INH is short for inhibition.

**Figure 13.**
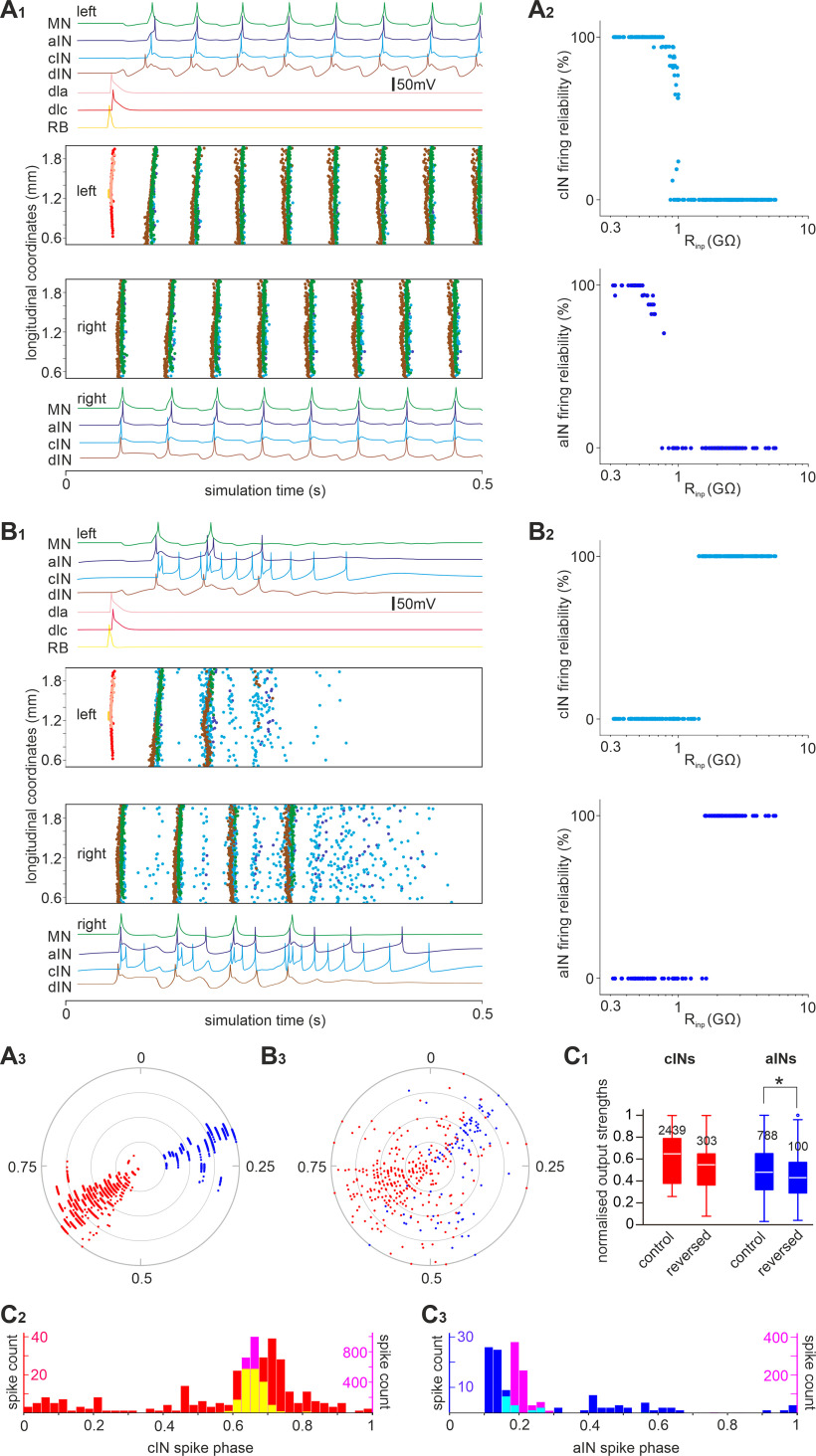
Simulating swimming using a network model including developing cINs/aINs. ***A_1_***, The control model network generates stable swimming rhythms when cIN/aIN input synaptic strengths decay exponentially with their R_inp−_ (functions derived from data in Figs. 6, 7). ***A_2_***, The cIN/aIN firing reliability is high for neurons with low R_inp_ and low when the R_inp_ is high in the network model in ***A_1_***. ***A_3_***, Circular plot showing the phase and strength of aIN and cIN spikes in the control simulation. ***B_1_***, The swimming rhythm breaks down in the reversed model in which the negative association between cIN/aIN R_inp_ and their input synaptic strengths are reversed. ***B_2_***, cINs/aINs with high R_inp_ fire reliably in the reversed model in ***B_1_***. ***B_3_***, Circular plot showing the phase and strength of aIN and cIN spikes in a reversed model simulation. ***C_1_***, Normalized aIN and cIN spike strengths in control and reversed models in ***A_3_*** and ***B_3_*** (numerals are numbers of spikes analyzed); *p < 0.05 (independent samples Mann–Whitney *U* test). ***C_2_*_,_*_3_***, cIN and aIN spike phase in control (pink) and reversed models (red for cINs, blue for aINs, in ***C_2_*** yellow shows the overlap of pink and red histograms and in ***C_3_*** cyan shows the overlap of pink and blue histograms). Color traces in ***A_1_***, ***B_1_*** show example activity of neurons of different categories during one simulation. Spiking events of individual neurons at different rostrocaudal coordinates in the whole network are shown as dots color matched with the recording traces. Firing reliability for each cIN/aIN in ***A_2_***, ***B_2_*** (dot) is calculated by dividing the number of spikes each neuron fires with the median number of spikes fired by all ipsilateral motoneurons between 0.1 and 0.6 s in the simulation (100% if >1). The radii of gray circles represent normalized output strengths for individual aIN and cIN spikes at 0.25, 0.5, 0.75 and 1, respectively in ***A_3_*** and ***B_3_***.

We have previously developed a detailed spiking neuronal network model of the tadpole spinal cord based on extensive anatomic and physiological data to simulate tadpole swimming (Roberts et al., 2014) in which all non-dIN neurons had identical ion channel composition and input resistances. Here, we modified cIN/aIN models to give them delayed firing properties as shown in Figure 5 (Sautois et al., 2007). In addition, we reassigned cIN and aIN input resistances (R_inp_) to match distributions in Figure 2. To describe the dependence between cIN/aIN R_inp−_ and the synaptic strengths (Figs. 6, 7), we fitted the data with exponentially decaying functions (Fig. 12). These were used to prescribe compound input synaptic conductance of cINs/aINs as functions of their R_inp_. We divided the compound conductance by the estimated number of presynaptic cells of each type to obtain unitary synaptic strengths and scaled these strengths to reproduce the firing reliability of cINs/aINs during swimming (control model). Stimulation of sensory neurons initiated reliable, alternating swimming activities of CPG neurons between the two body sides at frequencies between 15 and 19 Hz (Fig. 13*A1*; *n* = 100 connectomes). The cIN/aIN firing reliabilities qualitatively match experimental data in Figure 2 (Fig. 13*A2*). Thus, our simulations show incorporating developing cINs/aINs with high R_inp_ and weak input synaptic strengths in the swimming network does not destabilize the swimming rhythms.

Next, we used three approaches to destroy the negative association between R_inp_ and input synaptic strengths while maintaining the distributions of cIN/aIN R_inp_. In the first approach, we reversed the negative association between cIN and aIN R_inp_ and their input synaptic strengths by reversing the exponential correlations in the control model (Fig. 12, reversed model, red curves). In 100 simulations, sensory stimulation initiated brief swimming rhythms for one to four cycles, which broke down in all trials following tonic firing of cINs/aINs (Fig. 13*B1*). The natural recruitment pattern of cINs/aINs during the brief swimming rhythms was also reversed; that is, the majority of neurons with low R_inp_ fired few spikes, whereas those with higher R_inp_ spiked reliably.

**Figure 14.**
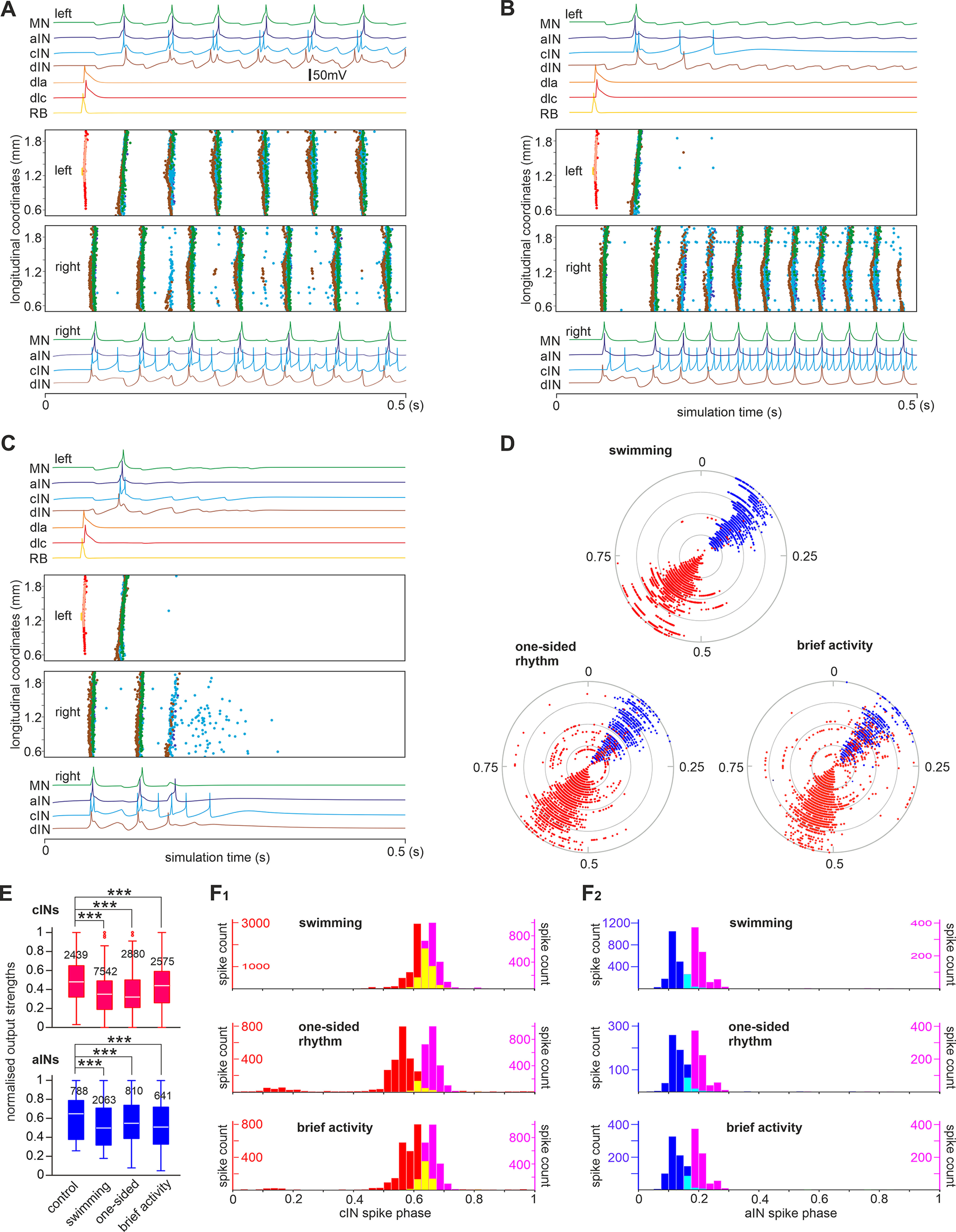
Simulation outcomes of the randomized model in response to sensory stimulation. ***A–C***, Swimming, one-sided rhythm, and brief activity that fails to persist. Color traces show example activity of neurons of different categories during one simulation. Spiking events of individual neurons at different rostrocaudal coordinates in the whole network are shown as dots color matched with recording traces. ***D***, Circular plots showing the phase and strength of aIN and cIN spikes in the randomized models with different outcomes. The radii of gray circles represent normalized output strengths for spikes at 0.25, 0.5, 0.75, and 1, respectively. ***E***, Normalized cIN and aIN spike strengths in control and reversed models in ***D*** (numbers represent spikes analyzed). ***F_1_*_,_*_2_***, cIN and aIN spike phase in control (pink) and reversed models (red for cINs, blue for aINs, In ***F_1_*** yellow shows the overlap of pink and red histograms and in ***F_2_*** cyan shows the overlap of pink and blue histograms); ***p < 0.001 (independent samples Mann–Whitney *U* tests).

The second approach was to randomly shuffle the cIN/aIN input synaptic strengths by assigning a random value from the data distribution in Figures 6 and 7 (randomized model). Among 100 simulations, stable swimming rhythms with alternating motoneuron firing was seen in 33 cases. In the remaining simulations, 47 cases showed one-sided rhythmic firing in CPG neurons with double the normal swimming frequency (one-sided activity), and 20 cases generated brief swimming rhythms that broke down after a few cycles, and the neuron membrane potentials converged to steady-state resting (Fig. 14, brief activity). Such a resting state is a simple stable output of the model in which all cells are inactive. The mechanisms leading to one-sided activity are clarified below.

Third, all cINs/aINs were assigned with high synaptic inputs regardless of their R_inp_ in 42 simulations in the mature inputs model. In 19 simulations, swimming rhythms broke down after a few swimming cycles (similar to Fig. 13*B1*). Reliable swimming was only seen in 2 simulations, while the remaining 21 simulations produced synchrony alternating with swimming in which CPG neurons showed frequent midcycle firing (similar to examples in Fig. 14*B* but with activities on both sides; data not shown).

We previously showed that dIN rebound firing from cIN inhibition was critical for swimming rhythm generation (Li et al., 2006; Soffe et al., 2009). In all models, most dINs fired reliably in a one-spike-per-cycle manner before the network activities stopped, but aIN and cIN firing varied. Therefore, the differences in aIN and cIN activities may have decided the network outcome. We analyzed the aIN/cIN spiking in the reversed and randomized models to identify changes that could potentially explain why swimming broke down. On one given side, dINs receive inhibition from ipsilateral aINs and contralateral cINs. Therefore, the spiking phase of aINs and opposite-side cINs was analyzed together with their output strengths in the network (see above, Materials and Methods). In the control model, aIN and cIN spiking was synchronized, and their phases were separated by nearly half a swimming cycle (788 aIN spikes, 0.2 ± 0.03; 2449 cIN spikes, 0.66 ± 0.03). In the reversed model, cIN spiking strengths remained similar to those of control, but the phase distribution broadened (independent samples Mann–Whitney *U* tests, *p <* 0.001; Fig. 13*B1–3*,*C1*,*C2*). aIN spiking strengths decreased, also with broader distribution than in control (independent samples Mann–Whitney *U* tests, *p <* 0.001; Fig. 13*A3*,*B3*,*C3*). In the randomized models, aIN and cIN spike strengths decreased in comparison to control models regardless of network outputs (all independent samples Mann–Whitney *U* tests, *p <* 0.001; Fig. 14*D*,*E*). aIN spiking was still synchronized, but the phase distribution peak shifted earlier in the swimming cycle (independent samples Mann–Whitney *U* tests, *p <* 0.001; Fig. 14*D*,*F*). In contrast, cIN spiking was more variable. Swimming rhythms persisted when the cIN spiking phase had low variance (0.66 ± 0.027, *n =* 2439). In the remaining cases when cIN spike timing was more variable and overlapped with aIN spiking, rhythmic activity stopped bilaterally (0.53 ± 0.135, *n =* 2575, *p <* 0.001) or unilaterally (0.58 ± 0.114, n= 2880, *p <* 0.001; both Levene’s test, Fig. 14*D–F*).

How could rhythm activity sustain only on one side in some of the randomized models with frequencies doubling that of normal swimming? We previously reported synchrony when both sides of the tadpole swimming circuit were active simultaneously with similar frequencies (Li et al., 2014; Ferrario et al., 2018b) in which cINs spiked immediately after dINs to evoke rebound firing more quickly and halved the cycle period. In the one-sided activity, the only source of inhibition-synchronous aIN firing also appeared shortly after dIN spiking (0.12 ± 0.025, *n =* 810; Fig. 14*B*), suitable for evoking dIN rebound firing nearly half a swimming cycle early and sustaining the one-sided rhythms in a similar manner (modeling in Ferrario et al., 2018a). In contrast, aIN spiking phase in the reversed model was more variable (0.28 ± 0.231, *n =* 100, *p <* 0.001; Levene’s test), which did not support one-sided rhythms.

These modeling results confirm that the swimming network incorporated with developing cINs/aINs with the experimentally derived properties still generate robust swimming rhythms, and negative association between the R_inp_ and input synaptic currents may be a critical factor in the uninterrupted integration of developing cINs/aINs in the swimming circuit. Indeed, when this association was destroyed, the firing of cINs/aINs became irregular and disrupted the swimming rhythm.

## Discussion

During differentiation, neurons acquire the correct neurochemical identity, extend dendrites and axons to target areas, and form connections with presynaptic and postsynaptic partners. They also need to express ion channels to tune electrical properties to suit their physiological roles. Here, we have analyzed the intrinsic firing and synaptic properties, anatomic features, and synaptic inputs/outputs of neurons in situ while simultaneously monitoring neuronal recruitment in swimming. We found the recruitment of inhibitory interneurons could be predicted by their R_inp_, an indicator for neuronal age in development (McCormick and Prince, 1987; Ramoa and McCormick, 1994; Zhang, 2004; Ehrlich et al., 2012). We identified that input synaptic strengths were critical in the order of recruitment.

Recruitment of motoneurons had been historically described by Henneman (1957), Henneman and Olson (1965), and Henneman et al. (1965a,b) as following the size principle. This proposes that larger mammalian spinal motoneurons with lower R_inp_ were only recruited at high motor strengths and decruited first when the muscle relaxed. In zebrafish larvae up to 5 d old, the motoneuron recruitment also follows size principle, but in both excitatory and inhibitory interneurons only recruitment orders by R_inp_ or dorsal-ventral positions are observed (McLean et al., 2007; Menelaou et al., 2022). Although interneurons with high R_inp_ are active at both slow and high swimming frequencies, those with low R_inp_ are only recruited at high frequencies. In adult zebrafish, the strong escape swimming and weaker explorative swimming are anatomically separate in that the latter is mediated by the caudal part of the spinal cord. Spinal motoneurons comprise four different pools, with recruitment more topographically determined by their location, electrical properties, and input synaptic currents rather than size or R_inp_ (Gabriel et al., 2011). Analyses of V0v excitatory interneurons with commissural projections in adult zebrafish revealed similar grouping and recruitment mechanisms (Bjornfors and El Manira, 2016). The topographic recruitment of neurons during larval zebrafish swimming was later shown to represent both an order of movement speed/strength and the temporal emergence of network components (Kimura et al., 2006; McLean and Fetcho, 2009). The recruitment of newly developed neurons in this case appears to expand the range of movement, that is, acquisition of weaker swimming in older larval fish (Berg et al., 2018).

The swimming frequency for stage 37/38 tadpoles ranges from 10 to 25 Hz (Roberts et al., 2010), narrower than the 20–100 Hz range of the larval zebrafish swimming (Saint-Amant and Drapeau, 1998; Muller and van Leeuwen, 2004). Unlike zebrafish larval swimming, the average tadpole swimming frequency does not vary much between episodes if the inter-episode resting periods remain similar (Zhang and Sillar, 2012). We find cINs/aINs with low R_inp_ fire more reliably in swimming, opposite to the recruitment order of inhibitory interneurons in larval zebrafish (McLean et al., 2007). For excitatory interneurons in zebrafish, those with high R_inp_ were often active at slow swimming but depressed during fast swimming (McLean et al., 2008; Kishore et al., 2014). The *En-1*-expressing V1 interneurons were found to selectively inhibit excitatory interneurons and motoneurons at high swimming frequencies (Kimura and Higashijima, 2019). Similar modular control of swimming speed is unlikely in stage 37/38 tadpoles as dINs fire reliably in a one-spike-per-cycle manner. In addition to swimming, the tadpole spinal circuit can also generate struggling rhythms, with motoneurons firing bursts of spikes, lower frequencies, and tail-to-head activity propagation (Soffe, 1993; Li, 2015). Our analyses show the recruitment of neither cINs nor aINs in struggling could be predicted by their R_inp_, against a possible motor-pattern-based recruitment regime.

We argue in stage 37/38 tadpoles that what we have described here is most likely a form of developmental integration of newly differentiated neurons into a functioning motor circuit to accommodate a growing, larger neuromuscular system. Before tadpoles reach stage 42, new interneurons are born continuously to add to the existing circuit (Dale et al., 1986; Roberts et al., 1987; 1988). The random recordings we made should include a mixture of more mature, early-born neurons and newly differentiated neurons with high R_inp_. The gradient of their firing reliability during swimming likely represents their progressive integration into the swimming circuit. At stage 42, swimming becomes more flexible following the addition of a new wave of small secondary neurons, neuromodulation, and refinement of neuromuscular innervation (Sillar et al., 1991; Zhang et al., 2011). Modular microcircuits enabling both weak and strong swimming similar to those in larval zebrafish may also exist.

Could cIN/aIN electrical properties explain the developmental recruitment or integration? Previous studies have identified some consistent changes in neuronal intrinsic properties during development (McCormick and Prince, 1987; Ramoa and McCormick, 1994; Zhang, 2004; McLean and Fetcho, 2009; Ehrlich et al., 2012). For example, with development, R_inp_ and time constant decrease, spike overshoot becomes higher, action potentials narrow, and firing thresholds become more negative. High R_inp_ and low firing thresholds make neurons more excitable, whereas the larger AHPs may lower their firing frequencies (Zhang, 2004; Ehrlich et al., 2012; Matschke et al., 2018). cINs/aINs with higher R_inp_ have lower firing thresholds, unsupportive of their lack of activity during swimming. Both types of neurons, however, show outward rectification (Ketchum et al., 1995; Maingret et al., 2002; Johnston et al., 2010), rendering neurons with higher R_inp_ more easily inhibited than excited and potentially suppressing their firing during swimming. In the meantime, less negative firing thresholds because of I_A_ does not necessarily reduce neuronal excitability as tonic excitation during swimming will inactivate I_A_ currents (Li, 2015).

The main factor that determines the developmental integration of cINs/aINs lies predominantly in their synaptic inputs, similar to what was observed in the prefrontal cortex (Zhang, 2004). Synaptic strengths are plastic in development because of changes in the postsynaptic receptor composition, presynaptic release probability, quantal response, or number of synaptic contacts (Mozhayeva et al., 2002; Isaac, 2003; Andreae et al., 2012; Herring and Nicoll, 2016). We did not find a correlation between EPSC receptor composition and R_inp−_, suggesting the absence of NMDAR-dependent plasticity in cIN/aIN inputs. The less reliable midcycle compound IPSCs in neurons with high R_inp−_ suggests low release probabilities from inhibitory synapses, but EPSCs are reliable. This may indicate dINs develop earlier in the circuit. In line with this, unitary IPSC but not EPSC strengths are negatively correlated with R_inp−_. The number of unitary inputs of both IPSCs and EPSCs, however, increases with decreasing R_inp−_, indicating that when neurons mature, they will receive inputs from more presynaptic partners. In our modeling, including developing cINs/aINs with the appropriate electrical and synaptic properties did not undermine swimming rhythm genesis. However, randomizing input synaptic strengths in cINs/aINs made neurons with high R_inp_ fire reliably and led to the breakdown of swimming rhythms. This was especially so when the relation between the R_inp_ of cINs/aINs and their input synaptic strengths was reversed. This supports the importance of our observed recruitment order and mechanisms in terms of maintaining circuit functions.

Transiently increased activity has been shown to play a role in the integration of newly differentiated neurons into local networks in mammalian olfactory bulb (Livneh et al., 2014) and hippocampus (Ge et al., 2006; Marin-Burgin and Schinder, 2012). cINs/aINs with higher R_inp_ did not exhibit any associated high activity in tadpoles, in line with findings in the developing zebrafish optic tectum (Heckman and Doe, 2021). Sensory (Livneh et al., 2014; Alvarez et al., 2016) or motor activity (Hall and Tropepe, 2018) also help to stabilize the connectivity of newborn neurons with mature circuits. Tadpole cINs/aINs normally do not receive direct sensory inputs but belong to the swimming CPG. It remains to be seen whether their integration into the swimming circuit is subject to similar activity-dependent plasticity.

Does neuronal morphologic growth match neuronal electrical properties and synaptic output? The lack of negative correlation between dendritic lengths and R_inp−_ suggests there may be a lag in leak potassium channel expression after dendritic extensions. cIN and aIN descending axon lengths are correlated with R_inp_ but the ascending branch lengths are not. The main axons for cINs/aINs are the ascending branches that develop earlier than the descending ones (Roberts et al., 1987, 1988), and they do not cross the midbrain/hindbrain border, potentially accounting for their lack of correlation with cIN/aIN R_inp_. Descending axons, in contrast, do not have a similar anatomic barrier unless they reach the caudal extremity. Their development may better coincide with the maturation of neuronal electrical properties. Meanwhile, because synaptic outputs of cINs/aINs are scaled with the R_inp_ of their postsynaptic partners, not with their own R_inp_, the molecular mechanism affecting synaptic strengths may be regulated by some target-derived factors, segregated from those controlling the maturation of electrical properties at somata and/or dendrites.

In summary, we have found several physiological and anatomic features of developing inhibitory interneurons that correlate with their participation/integration in swimming. Their recruitment is predictable by cellular input resistances but opposite to the order depicted by the motor-strength-based size principle. It is important to reveal how the integration process is regulated by various transcription and growth factors and whether such regulation has a critical time window.
